# Feasibility of functional precision medicine for guiding treatment of relapsed or refractory pediatric cancers

**DOI:** 10.1038/s41591-024-02848-4

**Published:** 2024-04-11

**Authors:** Arlet M. Acanda De La Rocha, Noah E. Berlow, Maggie Fader, Ebony R. Coats, Cima Saghira, Paula S. Espinal, Jeanette Galano, Ziad Khatib, Haneen Abdella, Ossama M. Maher, Yana Vorontsova, Cristina M. Andrade-Feraud, Aimee Daccache, Alexa Jacome, Victoria Reis, Baylee Holcomb, Yasmin Ghurani, Lilliam Rimblas, Tomás R. Guilarte, Nan Hu, Daria Salyakina, Diana J. Azzam

**Affiliations:** 1 Department of Environmental Health Sciences, Robert Stempel College of Public Health & Social Work, Florida International University, Miami, FL USA; 2 First Ascent Biomedical, Inc, Miami, FL USA; 3 Division of Pediatric Hematology Oncology, Department of Pediatrics, Nicklaus Children’s Hospital, Miami, FL USA; 4 Miller School of Medicine, University of Miami, Miami, FL USA; 5 Center for Precision Medicine, Nicklaus Children’s Hospital, Miami, FL USA; 6 Department of Biostatistics, Robert Stempel College of Public Health & Social Work, Florida International University, Miami, FL USA

**Keywords:** High-throughput screening, Functional genomics, Paediatric cancer

## Abstract

Children with rare, relapsed or refractory cancers often face limited treatment options, and few predictive biomarkers are available that can enable personalized treatment recommendations. The implementation of functional precision medicine (FPM), which combines genomic profiling with drug sensitivity testing (DST) of patient-derived tumor cells, has potential to identify treatment options when standard-of-care is exhausted. The goal of this prospective observational study was to generate FPM data for pediatric patients with relapsed or refractory cancer. The primary objective was to determine the feasibility of returning FPM-based treatment recommendations in real time to the FPM tumor board (FPMTB) within a clinically actionable timeframe (<4 weeks). The secondary objective was to assess clinical outcomes from patients enrolled in the study. Twenty-five patients with relapsed or refractory solid and hematological cancers were enrolled; 21 patients underwent DST and 20 also completed genomic profiling. Median turnaround times for DST and genomics were within 10 days and 27 days, respectively. Treatment recommendations were made for 19 patients (76%), of whom 14 received therapeutic interventions. Six patients received subsequent FPM-guided treatments. Among these patients, five (83%) experienced a greater than 1.3-fold improvement in progression-free survival associated with their FPM-guided therapy relative to their previous therapy, and demonstrated a significant increase in progression-free survival and objective response rate compared to those of eight non-guided patients. The findings from our proof-of-principle study illustrate the potential for FPM to positively impact clinical care for pediatric and adolescent patients with relapsed or refractory cancers and warrant further validation in large prospective studies. ClinicalTrials.gov registration: NCT03860376.

## Abstract (web summary)

In an observational study evaluating functional precision medicine in children and adolescents with relapsed or refractory solid and hematologic malignancies, it was feasible to provide personalized treatment recommendations to treating physicians on the basis of genomic profiling and ex vivo drug sensitivity testing within 4 weeks.

## Main

Cancer is the leading cause of disease-related death for children and teenagers in the United States. Despite improvements in survival for patients with cancers like acute lymphoblastic leukemia, progress for other high-risk, relapsed or refractory pediatric cancers remains challenging^1^. These patients typically have few established treatment options, in spite of advancements in standard therapy^2,3^. Genomics-guided precision oncology^4^ aims to provide pediatric and adolescent patients with matched treatments based on molecular changes in their tumors to improve survival and quality of life. The widespread availability of different sequencing approaches has resulted in multiple pediatric cancer precision medicine programs around the world such as the Zero Childhood Cancer Program in Australia, PROFYLE in Canada and iTHER in the Netherlands^5–7^. Despite the substantial clinical benefit, these trials revealed several constraints in using genomics-driven therapy only, particularly for cancers that lack actionable driver mutations and matched treatments, which is often the case in pediatric cancers are often driven by copy number alterations and/or gene fusions^8^. To overcome these limitations, recent trials like INFORM in Europe have begun to integrate functional ex vivo DST with genomics precision medicine to provide additional therapeutic options for patients who do not benefit from genomic profiling alone^9,10^. This approach, termed functional precision medicine (FPM), combines molecular profiling with direct ex vivo exposure of patient-derived tumor cells to drugs approved by the Food and Drug Administration (FDA). FPM expands available treatment options to patients who have exhausted standard-of-care treatment^11–13^. The feasibility and clinical efficacy of FPM for adults with hematological cancers have been investigated in two recent FPM trials, in Finland and Austria^14,15^, with both of these independent studies demonstrating that the integration of molecular profiling and high-throughput DST provides clinical benefit to these patients and provides robust data for further translational research. However, interventional FPM trials have so far exclusively addressed patients with hematological cancers owing to technical challenges regarding DST in solid malignancies and, until now, have solely enrolled adults. Critically, prospective FPM studies for pediatric patients with cancers are lacking.

The aim of our study was to determine the feasibility of combining ex vivo DST with targeted genomic profiling to generate FPM data for pediatric patients with relapsed or refractory cancers. We present results from a prospective, non-randomized, single-arm observational feasibility study (ClinicalTrials.gov registration: NCT03860376) in children and adolescents with relapsed or refractory solid and hematological cancers. Data from tumor panel profiling and functional ex vivo DST of up to 125 FDA-approved drugs were generated. We report successful outcomes for our primary objective of returning data to an FPM tumor board (FPMTB) in a clinically relevant timeframe. We also report, as our secondary objective, comparisons between the clinical outcomes of FPM-guided treatment and the patients' previous regimens, as well as between the outcomes of FPM-guided treatment and treatment of physician’s choice (TPC). Our study demonstrates the feasibility and clinical utility of an FPM approach to prospectively identify treatment options for patients with advanced solid and hematological malignancies, regardless of tumor type, particularly for high-risk cancers such as those affecting pediatric and adolescent patients.

## Results

### Patient characteristics and study design

Between 21 February 2019 and 31 December 2022, we conducted a prospective study at Nicklaus Children’s Hospital (Miami, Florida, USA). The primary objective was to determine the feasibility of returning FPM results to an FPMTB, which included treating physicians, in a clinically actionable timeframe (within 4 weeks) to inform treatment decisions. We considered this objective met if we returned treatment options to at least 60% of enrolled patients. The secondary study objective was to compare clinical outcomes of enrolled patients who underwent FPM-guided treatment to both the outcomes of their previously received treatments and those of patients who received TPC. All patients had objective response and progression-free survival (PFS) from their prior regimen recorded at the time of enrollment for comparison against study outcomes.

Treatments were not given as part of the study. Separate consents were required for any selected treatment regimens. All decisions regarding treatment regimens were made by the treating physician and, although it could be influenced by the FPM data, the final treatment selection for each patient was at the sole discretion of the treating physician based on their experience and expertise.

We enrolled a total of 25 pediatric and adolescent patients with recurrent or refractory solid (*n* = 19; 76%) or hematological (*n* = 6; 24%) malignancies. Twenty-three of 25 patients were enrolled from Nicklaus Children’s Hospital, one patient from St. Mary’s Medical Center at Palm Beach Children’s Hospital and one patient from Oregon Health and Science University (Supplementary Table; see Testing and demographics).

**Fig. 1:**
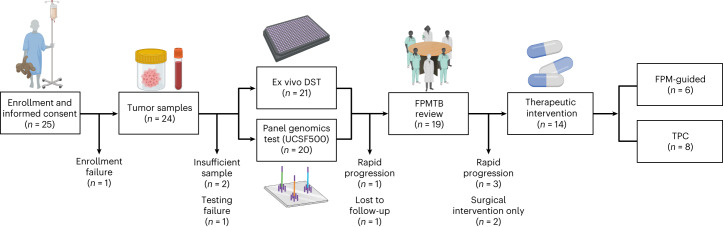
Flow diagram showing FPM workflow. FPM workflow including patient enrollment, sample collection, functional ex vivo drug sensitivity testing and molecular tumor profiling, and report delivery to the FPMTB for clinical decision-making. Numbers at each exit and endpoint represent patient numbers. Created with BioRender.com.

Patients were enrolled after exhausting standard-of-care options, irrespective of cancer type. Solid tumor biopsies (*n* = 1) or resections (*n* = 17), or hematological cancer samples (*n* = 6) were obtained for ex vivo DST and genomic panel profiling (using the UCSF500 test). The median time from sample collection at the clinic to arrival in the processing laboratory was less than 48 h for all patients. DST was successfully performed on 21 out of 24 patients (88%) who provided tumor tissue samples. UCSF500 profiling was performed on 20 out of 24 patients (83%). Figure 1 describes patients who were removed from the study owing to enrollment failure (*n* = 1), insufficient sample size for both DST and genomic profiling (*n* = 2) and unsuccessful DST (*n* = 1). FPM results from two patients were not discussed by the FPMTB owing to loss at follow-up or rapid disease progression. Thus, 19 out of 25 enrolled patients (76%) completed both DST and genomic profiling and had the results reported to an interdisciplinary FPMTB for review, surpassing our original objective of 60% of enrolled patients (*P* < 0.0001, 95% confidence interval (CI) 0.5487–0.9064). Of the 19 patients whose results were discussed, tumors from three patients progressed too rapidly for treatment and two patients underwent surgical intervention only, with 14 patients receiving therapeutic interventions. Overall, six patients received FPM-guided therapy, and eight patients received TPC (Fig. 1).

Baseline demographics for all enrolled patients are shown in Table 1. The median age of the patient cohort was 10 years. Of the enrolled patients, 40% were female (10 patients) and 60% were male (15 patients), with a slightly lower female-to-male ratio than the national 1:1.1 incidence ratio of pediatric cancers^1^. Patient enrollment approximated the diverse population of pediatric patients with cancer of the Miami-Dade County area from which patients were accrued^16^. Of those enrolled, three patients (12%) were Black or African American, 17 patients (68%) were Hispanic (16 white Hispanic (64%), one mestizo (4%)) and five patients (20%) were white.

**Table 1:** Characteristics of enrolled patients with pediatric cancer

Characteristics	Count	%
All	25	100
Sex		
Female	15	60
Male	10	40
Age, median (range) (years)	10 (0.81–21)	
Race		
White	21	84
Black or African American	3	12
Other (mestizo)	1	4
Ethnicity		
Hispanic	17	68
Non-Hispanic	8	32
Previous therapy lines, median (range)	3 (2–6)	
Disease type		
Acute lymphoblastic leukemia	3	12
Acute myeloid leukemia	3	12
Astrocytoma	1	4
Ependymoma	1	4
Ewing sarcoma	4	16
Glioblastoma multiforme	1	4
Medulloblastoma	1	4
Malignant rhabdoid tumor	1	4
Neuroblastoma	1	4
Osteosarcoma	4	16
Rhabdomyosarcoma	4	16
Wilms tumor	1	4

In addition, enrolled patients had a variety of pediatric cancer indications, encompassing 12 different pediatric malignancies: three acute lymphoblastic leukemias (ALLs), three acute myeloid leukemias (AMLs), one astrocytoma (AST), one ependymoma, four Ewing sarcomas (EWSs), one glioblastoma (GBM), one malignant rhabdoid tumor (MRT), one medulloblastoma, one neuroblastoma, four osteosarcomas, four rhabdomyosarcomas (RMS), and one Wilms tumor. All hematological cancers were leukemias (12% each); solid malignancies consisted of sarcoma (48%), central nervous system tumors (20%) and kidney cancers (8%). Genomics testing and DST were successfully performed across all cancer types, with only one EWS sample failing DST (Supplementary Table; see Testing and demographics).

### Patient-derived tumor cultures and DST

**Fig. 2:**
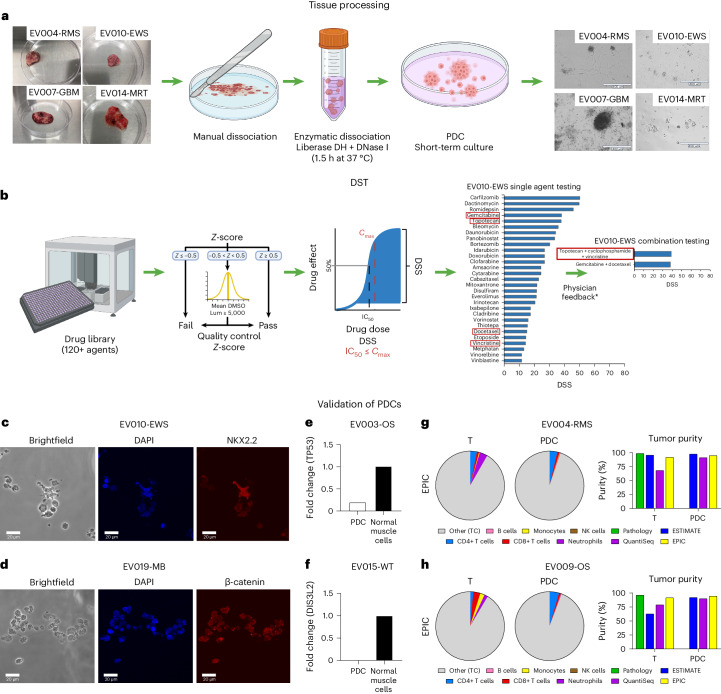
Dissociation of tumor tissue workflow, DST analysis and validation of patient-derived tumor cultures. **a**, Tissue processing and derivation of short-term PDCs, including representative images of received tissues (left) and derived PDCs (right) from EV004-RMS, EV007-GBM, EV010-EWS and EV014-MRT. **b**, Ex vivo DST using a library of more than 125 FDA-approved drugs, post-endpoint quality control process based on *Z*-prime scores, IC_50_ and DSS analysis, and representative results from single agent testing for EV010-EWS followed by physician-selected drug combinations (if additional PDC material remained). Lum, luminescence. * indicates physician feedback guided selection of tested drug combinations. The slim red borders around single agents on the left indicate those included in combination testing, The thick red border on the right indicates the final drug combination used for the patient. **c**,**d**, Molecular characterization and validations of PDCs assessed by immunofluorescence detection of pathology-defined markers in EV010-EWS (**c**) and EV019-MB (**d**). Immunofluorescence images of one independent experiment (due to limited PDC material). **e**,**f**, Analysis of RT–qPCR to confirm loss of *TP53* transcripts in EV003-OS (**e**) and *DIS3L2* transcripts in EV015-WT (**f**). **g**,**h**, Immune cell type deconvolution and tumor purity analysis from tumor tissue at enrollment (T) and PDC in EV004-RMS (**g**) and EV009-OS (**h**) using bulk RNA-seq deconvolution tools EPIC, ESTIMATE and quanTIseq (right panel). Representative pie charts present EPIC deconvolution results. TC, tumor cell. Portions of panels **a** and **b** were created with BioRender.com.

**Extended Data Fig. 1:**
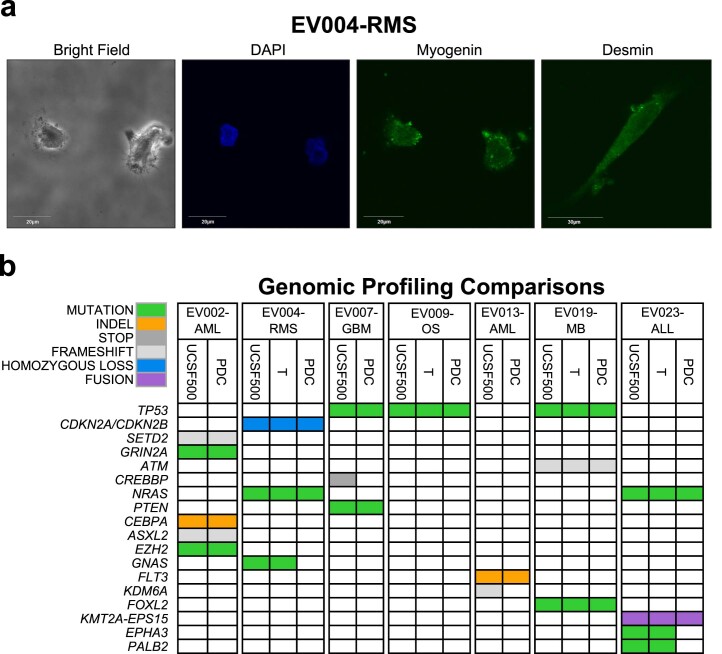
Immunofluorescence and Genomic Profiling Validation of PDCs. (**a**) Immunofluorescence analysis confirming the presence of pathology markers myogenin and desmin in EV004-RMS. Images taken at 90x using a laser scanning confocal microscope (Fluoview FV10i, Olympus) utilizing the FV10 image software. Representative images of one independent experiment due to limited PDC material. (**b**) Comparison of genomic alterations detected in UCSF500 tumor panel profiling with genomic profiling of original tumor sample at enrollment (T) and PDC at time of DST for EV002-AML, EV004-RMS, EV007-GBM, EV009-OS, EV013-AML, EV019-MB, EV023-ALL. Color code on the left indicates type of variant identified from UCSF500 profiling.

**Extended Data Fig. 2:**
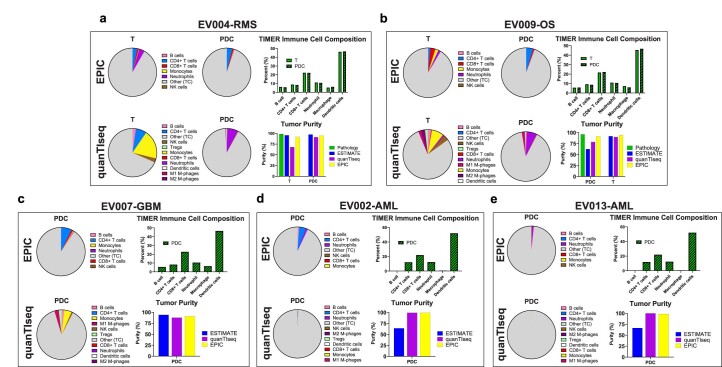
RNA-seq and Tumor Purity Validation of PDCs. Immune cell type deconvolution and tumor purity analysis was done from original tissue (T) and PDCs (when available). a) Analysis of EV004-RMS. Bulk RNA-seq was deconvoluted using the analysis tools EPIC (Top Left, T and PDC) and quanTIseq (Bottom Left, T and PDC). Immune cell composition (T and PDC) was analyzed using TIMER (Top Right). Tumor purity analysis was done using pathology analysis (T, in green), ESTIMATE (T and PDC, in blue), quanTIseq (T and PDC, in purple), and EPIC (T and PDC, in yellow). A similar approach was used in b) EV009-OS, c) EV007-GBM, d) EV002-AML and e) EV013-AML.

The DST component of the FPM workflow, shown in Fig. 2, consisted of three main steps. First, we carried out tissue processing and derivation of short-term patient-derived tumor cultures (PDCs) (Fig. 2a). Interestingly, most PDCs from solid tumor tissue samples grew in culture as a mix of free-floating or semi-adherent 3D clusters and individual adherent cells (see representative brightfield images of PDCs in Fig. 2a, right panel). Second, DST was performed on PDCs (Fig. 2b) using a library of up to 125 FDA-approved agents including 40 formulary drugs from Nicklaus Children’s Hospital, 47 non-formulary FDA-approved anti-cancer drugs, therapies in phase III or IV pediatric cancer clinical trials, and additional non-cancer agents that have been investigated for potential repurposing as anticancer treatments (Supplementary Table; see Drug list). PDCs were treated with drugs for 72 h, which is a standard timepoint for primary cell DST^17^. Within this timeframe, even slow-acting epigenetic drugs have shown efficacy according to our data^12,18^. *Z*-prime scores and luminescence values from wells with untreated cells were used as quality control measures for individual assay plates^9,19^. Only data from assay plates that passed quality control were analyzed and reported (Fig. 2b, middle panel). Drug sensitivity scores (DSSs) and half-maximum inhibitory concentration (IC_50_) values were derived from dose–response data. DSS is based on normalized dose–response area under the curve (AUC) and are often used in FPM or PDC-based studies^14,20,21^. Drugs were ranked for efficacy based on the DSS and recommended to the FPMTB for treatment if the IC_50_ was less than or equal to the maximum clinically achievable plasma concentration of the drug (*C*_max_) demonstrated to be safe and effective according to pharmacokinetic data reported in human clinical trials^22^. As monotherapy is not generally effective in treating relapsed pediatric cancers, physician-requested combination treatments were subsequently tested when additional PDC material was available (Fig. 2b, right panel). Final treatment plans were developed at the discretion of the treating clinicians and accounted for drug availability, insurance coverage, the patient’s previous treatment history and the physician’s own knowledge and expertise. Last, molecular characterization of PDCs was performed at the time of DST to confirm that PDCs maintained specific characteristics from original samples at time of enrollment, as described in the Methods. Validations were performed using different approaches. When possible, the presence of pathological markers reported in pathology reports was confirmed in PDCs using immunofluorescence, as demonstrated in representative images of PDCs from EV010-EWS, EV019-MB and EV004-RMS confirming NKX2.2, beta-catenin, and desmin and myogenin expression, respectively (Fig. 2c,d and Extended Data Fig. 1a). Specific genomic alterations mentioned in UCSF500 profiling, such as loss of *TP53* and *DIS3L2* transcripts in EV003-OS and EV015-WT, respectively, were also confirmed using quantitative PCR with reverse transcription (RT–qPCR) (Fig. 2e,f). Genetic stability in PDCs was established by comparing UCSF500-identified variants reported for the tumor at the time of enrollment with whole exome sequencing and/or whole transcriptome sequencing data (Extended Data Fig. 1b). In addition, multicellular composition analysis was performed on tumors at the time of enrollment and on PDCs for a subset of samples using immune cell type RNA sequencing (RNA-seq) deconvolution, as previously described^9^. The analysis of cell populations demonstrated a mean tumor cell content of 90% or higher at the time of DST (Fig. 2g,h and Extended Data Fig. 2). Importantly, the heterogeneity of tumors was conserved under our established culture conditions, as evidenced through RNA-seq and deconvolution approaches. Overall, PDC validation analyses revealed similarity between tumor samples and corresponding PDCs, as evident in the maintenance of relevant molecular driver aberrations and preservation of tumor cell content, indicating our ability to establish culture models with mixed cell populations (including immune cells) that closely resemble the multicellular compositions present in the respective tumor. A list of all validation tests performed on PDCs is provided in the Supplementary Table (see Culture validation experiments).

### FPM is feasible in a clinically actionable timeframe

**Fig. 3:**
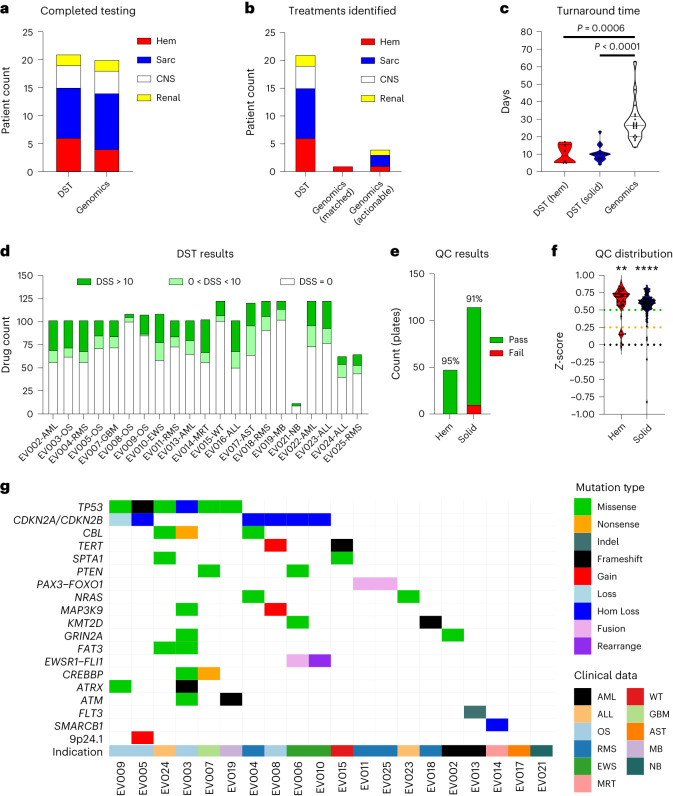
FPM workflow is feasible and actionable in a clinically relevant timeframe. **a,** Results returned from patient sample testing through DST and genomic profiling, distributed by cancer type. CNS, central nervous system; Hem, hematological; Sarc, sarcoma. **b**, Distribution of patients with reported therapeutic options identified through DST, identified by genomics as an approved therapy matching the patient’s cancer type (Matched) and identified by genomics as an approved therapy in other cancer types (Actionable). **c**, Distribution of turnaround time in days for DST of hematological cancer samples and solid cancer samples, as well as UCSF500 genomics panel assays. *P* values determined by adjusted Kruskal–Wallis test (*P* < 0.0001). **d**, Distribution of single agent DSS for each patient (ineffective, DSS = 0 (white); moderately effective, 0 < DSS ≤ 10 (light green); effective, DSS > 10 (dark green)). **e**, Number and percent of DST plates that passed quality control analysis for hematological and solid cancers. QC, quality control. **f**, *Z*-prime scores of quality control from DST plates for hematological and solid cancers. *P* values determined by two-sided one-sample Wilcoxon tests. Hem, *P* = 0.0045; solid, *P* = 0.00001. **P < 0.01, ****P < 0.0001. **g**, Genomic landscape of variants identified through genomic tumor panel profiling using UCSF500. Genes with alterations in two or more patient samples or alterations with matched therapies are reported. Hom, homozygous.

Actionable treatment recommendations were returned for 21 out of 25 enrolled patients using DST (84%), with 20 out of 25 patients also receiving results from genomics profiling (Fig. 3a). Five of those 20 patients (25%) had an actionable treatment recommendation based on genomic variants, and only one of those five patients received a recommendation for cancer-matched therapy^23,24^. This proportion was significantly less than DST recommendations, which identified treatment options in 21 of 21 patients (100%) (*P* < 0.0001, 95% CI) (Fig. 3b and Supplementary Table (see the actionable panel sequencing results and complete panel sequencing results)). These results demonstrate the benefit of DST in providing additional treatment options to pediatric patients with cancer compared to genomic profiling alone.

The turnaround time for DST results significantly outpaced the return of genomic profiling data. Following sample receipt, the median time for reporting DST results to the FPMTB was 9 days for hematological cancers (range, 5–17 days) and 10 days for solid tumors (range, 4–23 days) (Fig. 3c), significantly faster than the median turnaround time of 26.5 days (range, 14–63 days) for UCSF500 profiling (Fig. 3c). Rapid turnaround time enabled the FPMTB to promptly discuss each patient using functional DST data alone, with treatments modified when genomics results became available, if necessary and possible. For pediatric and adolescent patients with aggressive disease, the speed at which recommendations were made was critical for enabling guided therapeutic decision-making.

We considered DSS > 10 as effective, 0 ≤ DSS < 10 as moderately effective and DSS < 0 as ineffective. The analysis of DST results showed that the median number of effective and moderately effective drugs was 21 (range, 3–36) and 12 (range, 0–32), respectively (Fig. 3d and Supplementary Table (see DST testing results)). Accordingly, all patients had a minimum of three effective treatments identified. Furthermore, the median percentage of effective and moderately effective tested drugs was 21% (range, 4–35%) and 12% (range, 0–26%), respectively.

**Extended Data Fig. 3:**
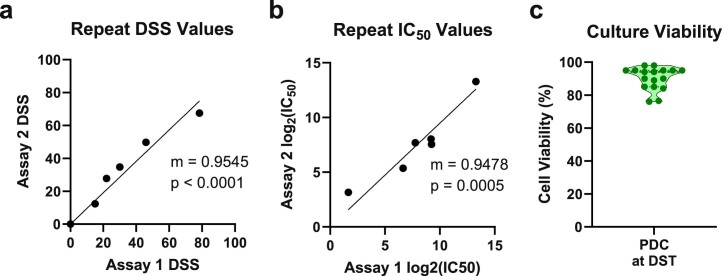
Additional Repeatability and Viability Metrics. (**a**) Correlation of DSS values from repeated assays (p = 0.00001). m represents the slope of the linear regression line; p value is from two-sided Pearson correlation analysis. b) Correlation of log2(IC50) values from repeated assays. m represents the slope of the linear regression line; p value is from two-sided Pearson correlation analysis. c) Percent cell viability of the PDCs at the time of DST assay.

At study completion, 96% (46 out of 48) of hematological cancer assay plates and 91% (105 out of 115) of solid cancer assay plates passed internal quality control, resulting in an overall quality control pass rate of 93% (151 out of 168) (Fig. 3e and Supplementary Table (see Z’ statistics)). The median *Z*-prime score was significantly above the 0.5 quality control cutoff for both hematological (*P* = 0.0045) and solid (*P* < 0.0001) cancer assays (Fig. 3f). Additionally, there was high correlation (*P* < 0.0001) between DSS and IC_50_ results in repeated DSTs (Extended Data Fig. 3a,b and Supplementary Table (see DST repeat data)). Median cell viability at the time of DST was 94% (range, 76–98%) (Extended Data Fig. 3c).

Diverse genomic profiles were identified through UCSF500 profiling. Of the genomic variants discovered, six were found in tumors for more than three patients, including *TP53* mutations (30%), *CDKN2A/B* loss (25%) and *CBL* variants (15%). *CBL* variants were of particular interest, as they have not been previously reported in pediatric cancers but have been established in a variant-associated tumor predisposition syndrome (Fig. 3g)^25^. Additionally, other genetic variants frequently found in cancers were identified, including *MYC* or *MYCN* mutations (one amplification each, 5%), and disease-specific gene fusions, including *PAX3-FOXO1* in alveolar RMS (two out of two patients, 100%) and *EWSR-FLI1* fusions in EWS (two out of four patients, 50%) (Fig. 3g). The sole actionable mutation matched to a patient’s cancer type was a *FLT3-ITD* mutation identified in one out of two sequenced patients with AML (50%) (Fig. 3g). Other actionable genomic variants included *SMARCB1* loss (one patient, 5%), amplification of 9p24.1, which includes *PD-L1*, *PD-L2* and *JAK2* (one patient, 5%), and an *NRAS* p.Q61K mutation (two patients, 10%) (Fig. 3g), although none provided treatment recommendations that matched the patients’ cancer types (Supplementary Table; see Actionable panel sequencing results).

### Patients guided by FPM have improved clinical outcomes

**Fig. 4:**
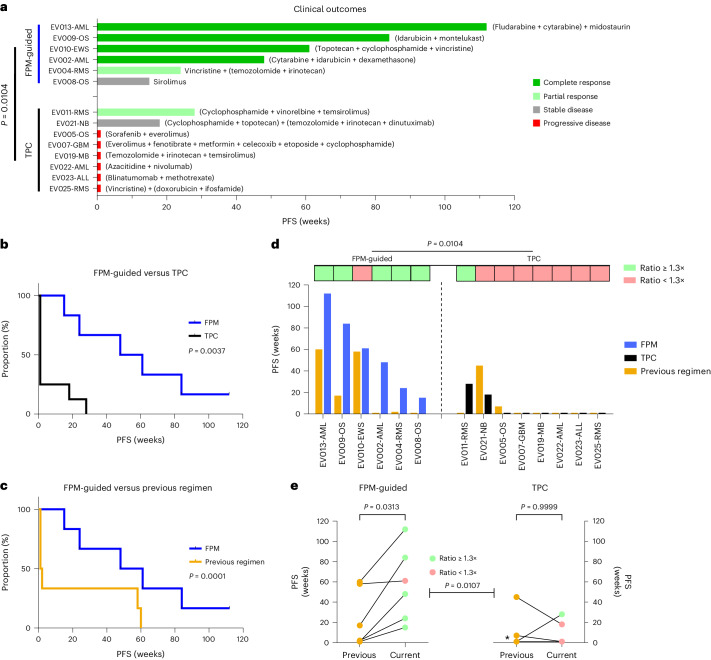
FPM-guided therapies provide significant clinical benefit in patients with refractory or relapsed pediatric cancer. **a**, Swimmer plot illustrating patient best objective response and PFS to treatments assigned following FPMTB review, grouped by FPM-guided and TPC-treated patients. Agents beside each patient represent treatments given during the study. *P* value determined by two-sided Barnard’s test. **b**, Comparison of PFS in the TPC-treated and FPM-guided cohorts. *P* value determined by logrank test analysis of Kaplan–Meier survival data. **c**, Comparison between the PFS of the trial regimen and the PFS of the patient’s previous regimen in the FPM-guided cohort. *P* value is from two-sided Cox proportional hazards test of paired survival data. **d**, Comparison of PFS from the previous regimen (orange in bar graph) and trial regimens for both FPM-guided (blue in bar graph) and TPC (black in bar graph) cohorts, with indications for patients with a PFS ratio of ≥1.3× (light green boxes above indicated patients) and <1.3× (light red boxes above indicated patients). *P* value determined by two-sided Barnard’s test analysis of occurrences of PFS ratio of ≥1.3×. **e**, Difference in PFS of the previous regimens and trial regimens for FPM-guided (left) and TPC-treated (right) cohorts. Asterisk, five patients who received TPC and had the same previous and trial regimen PFS. *P* values for each cohort determined by two-sided paired Wilcoxon test. *P* value between cohort determined by two-sided Mann–Whitney *U*-test of PFS ratio values. Light green dots indicate patients with a PFS ratio of ≥1.3× (top), light red dots indicate patients with a PFS ratio of <1.3×, and orange dots indicate the PFS of the previous regimen for both cohorts.

All patients enrolled in our study received at least two lines of previous treatments (median three lines; range, 2–6). Hence, standard-of-care was exhausted for all patients before enrollment. Treatment decisions were made by the interdisciplinary FPMTB for each individual patient. Of the 14 patients who received therapeutic interventions, six patients (43%) received subsequent FPM-guided treatments and eight (57%) received non-guided TPC (Fig. 4a). Characteristics of all patients who received therapeutic interventions are listed in Table 2.

**Table 2:** Description of previous treatments, clinically significant mutations and outcomes of patients receiving therapeutic intervention during the study

Patient ID	General diagnosis	Sample type	Sex	Age (years)	Previous therapy lines	Previous treatment	Previous objective response	Previous PFS (weeks)	Clinical mutations	FPM-guided	Additional intervention	Study treatment	Current objective response	Current PFS (weeks)
EV013	AML	Peripheral blood	M	5	3	Cyclophosphamide + busulfan + sorafenib	Complete response	60	*FLT3-ITD, KDM6A*	Yes	Allogeneic bone marrow transplant	Fludarabine + cytarabine, midostaurin	Complete response*	112
EV009	OS	Excised tumor	F	9	3	High-dose ifosfamide + larotrectinib	Stable disease	17	*CDKN2A/B, TP53, ATRX, ZFHX4*	Yes	Resection	Idarubicin + montelukast	Complete response	84
EV010	EWS	Excised tumor	M	21	5	Irinotecan + temozolomide + vigil vaccine	Complete response	58	*CDKN2A/B, EWSR1-FLI1, ESR2, AD21, TOP2A*	Yes	None	Topotecan + cyclophosphamide + vincristine	Complete response	61
EV002	AML	Peripheral blood	M	16	2	Cytarabine	Progressive disease	N/A	*CEBPA, ASLX2, SETD2*	Yes	None	Cytarabine + idarubicin + dexamethasone	Complete response	48
EV004	RMS	Excised tumor	F	7	3	Pazopanib + nivolumab	Progressive disease	2	*CDKN2A/B, GNAC, NRAS*	Yes	None	Vincristine, temozolomide + irinotecan	Partial response	24
EV008	OS	Excised tumor	F	17	2	Ifosfamide + mifamurtide	Progressive disease	N/A	*CDKN2A/B, TERT, CCDN3*	Yes	Resection	Sirolimus	Stable disease	15
EV011	RMS	Excised tumor	M	4	3	Irinotecan + cyclophosphamide	Progressive disease	N/A	*BCOR, PAX3-FOXO1, SMARCA4*	No	None	Cyclophosphamide + vinorelbine + temsirolimus	Partial response	28
EV021	NB	Excised tumor	F	4	2	Temozolomide + irinotecan + dinutuximab	Stable disease	45	None	No	None	Cyclophosphamide + topotecan, temozolomide + irinotecan + dinutuximab	Stable disease	18
EV005	OS	Biopsy of metastatic nodule	F	7	2	Ifosfamide	Complete response	7	*CDKN2A/B, MYC, TP53, 9p24.1, NOTCH3, NF1*	No	None	Sorafenib + everolimus	Progressive disease	N/A
EV007	GBM	Excised tumor	M	11	2	Bevacizumab + lomustine	Progressive disease	N/A	*CREBBP2, TP53, PTEN*	No	Radiation	Everolimus + fenofibrate + metformin + celecoxib + etoposide + cyclophosphamide	Progressive disease	N/A
EV019	MB	Excised tumor	F	12	2	Cisplatin + vincristine + cyclophosphamide	Progressive disease	N/A	*TP53, GLI2, MYCN, ATM*	No	None	Temozolomide + irinotecan + temsirolimus	Progressive disease	N/A
EV022	AML	Bone marrow aspirate	M	1.94	5	Clofarabine + cytarabine	Progressive disease	N/A	N/A	No	None	Azacitidine + nivolumab	Progressive disease	N/A
EV023	ALL	Peripheral blood	M	0.97	2	Vincristine + daunorubicin + cytarabine	Progressive disease	N/A	*NRAS, KMT2A-EPS15, EPHA3, PALB2*	No	None	Blinatumomab + methotrexate	Progressive disease	N/A
EV025	RMS	Excised tumor	F	8	2	Vincristine	Progressive disease	N/A	*PAX3-FOXO1*	No	None	Doxorubicin + ifosfamide + mesna	Progressive disease	N/A

ALL, acute lymphoblastic leukemia; AML, acute myeloid leukemia; EWS, Ewing sarcoma; GBM, glioblastoma multiforme; MB, medulloblastoma; NB, neuroblastoma; OS, osteosarcoma; RMS, rhabdomyosarcoma. *indicates the patient received a bone marrow transplant.

Remarkably, five out of six FPM-guided patients (83%) achieved an objective response (partial response or better), and all FPM-guided patients achieved stable disease or better as their best overall response (Fig. 4a). By contrast, only one of eight TPC-treated patients (13%) achieved an objective response, and six of those eight (75%) continued to experience progressive disease (Fig. 4a). Thus, the FPM-guided cohort experienced a significantly improved objective response rate (ORR) compared to that of the TPC-treated cohort (*P* = 0.0104, Barnard’s test; Fig. 4a). Importantly, PFS in the FPM-guided cohort was significantly longer than that of both of their matched previous regimens (*P* = 0.0001, Cox proportional hazards test; Fig. 4c) and the TPC cohort (*P* = 0.0037, logrank test; Fig. 4b).

**Extended Data Fig. 4:**
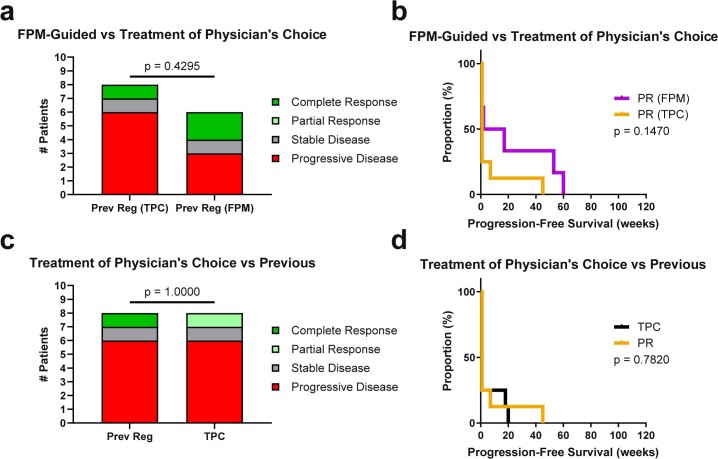
Additional Outcome Analysis. (**a**) Overall response distributions of therapeutic response prior to study enrollment in TPC cohort and FPM-guided patients. P values from two-sided Barnard’s test comparing ORR. (**b**) Kaplan-Meier survival curves of previous regimen PFS in TPC and FPM-guided patients. P values from Logrank test analysis of PFS data. (**c**) OR distributions of previous versus current regimen in TPC cohort. P values from two-sided McNemar’s Paired test comparing ORR. (**d**) Kaplan-Meier survival curves of the previous versus current regimen PFS in TPC cohort. P values from two-sided Cox Proportional Hazard test analysis of paired PFS data. PR = Previous Regimen, TPC = Treatment of Physician’s Choice, FPM = FPM-guided.

Owing to the small, heterogenous nature of our study cohort, we assessed a now commonly used metric in precision oncology studies: the ratio of PFS between the current and previous regimens (PFS ratio), whereby a patient’s clinical outcome serves as its own control and a PFS ratio of ≥1.3 is considered a positive outcome^15,26–29^. Patients in both treatment cohorts presented with similarly poor outcomes from previous regimens, with no significant differences in ORR (*P* = 0.4295; Extended Data Fig. 4a) or PFS (*P* = 0.1470; Extended Data Fig. 4b) between cohorts.

Interestingly, significantly more FPM-guided patients achieved a PFS ratio of ≥1.3× (median 8.5×; range, 1.05–48) than TPC-treated patients (median 1×; range, 0.14–28) (*P* = 0.0104, Barnard’s test; Fig. 4d), demonstrating that patients were more likely to have improved PFS when treatments were guided by FPM (*P* = 0.0313, paired Wilcoxon test; Fig. 4e) while TPC patients were not (*P* = 0.9999, paired Wilcoxon test; Fig. 4e). Patients receiving TPC did not demonstrate any significant differences in ORR (*P* = 1.0000; Extended Data Fig. 4c) or PFS (*P* = 0.7820; Supplementary Fig. 4d) between current and previous regimens. These data, therefore, indicate that FPM-guided treatment leads to better outcomes than TPC in pediatric patients with cancer.

**Extended Data Fig. 5:**
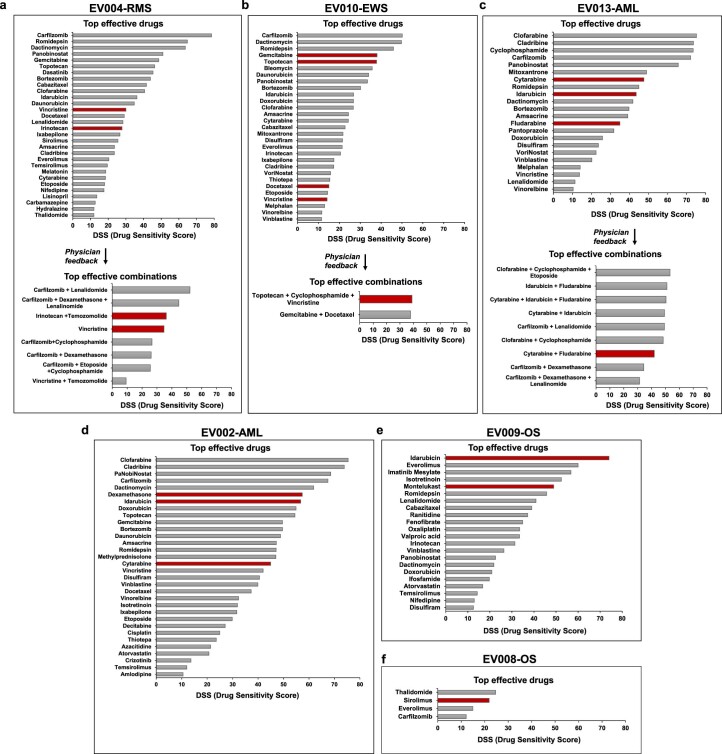
Top effective drugs for FPM-guided patients. DSS for top effective single agents (top) and top effective physician-requested combinations (bottom), defined as DSS > 10, are shown for (**a**) EV004-RMS, (**b**) EV010-EWS, (**c**) EV013-AML, (**d**) EV002-AML, (**e**) EV009-OS, and (**f**) EV008-OS. Drugs and combinations selected for therapy by treating physician marked in red.

Treatments guided by FPM were selected based on the patient’s individual FPM data. Although these treatments were often similar to standard-of-care options, for these patients the physicians relied on DST results, reflected in the DSS waterfall plots, to select the drugs used for treatment for each patient (Extended Data Fig. 5 and Supplementary Table – DST testing results, DST combination results). Some of these agents, such as statins and montelukast, have been investigated for potential repurposing as anticancer treatments^30,31^. Montelukast, in particular, was used in EV009-OS owing to its low toxicity, easy availability and efficacy in DST. When DST of drug combinations resulted in comparable DSSs, physicians generally selected the combination with lower expected toxicity based on previous experience. Thus, the FPM cohort largely received standard and readily accessible chemotherapy agents, establishing the utility of our functional testing platform in repurposing and prioritizing approved existing drugs to overcome resistance in heavily treated progressive cancers.

**Extended Data Fig. 6:**
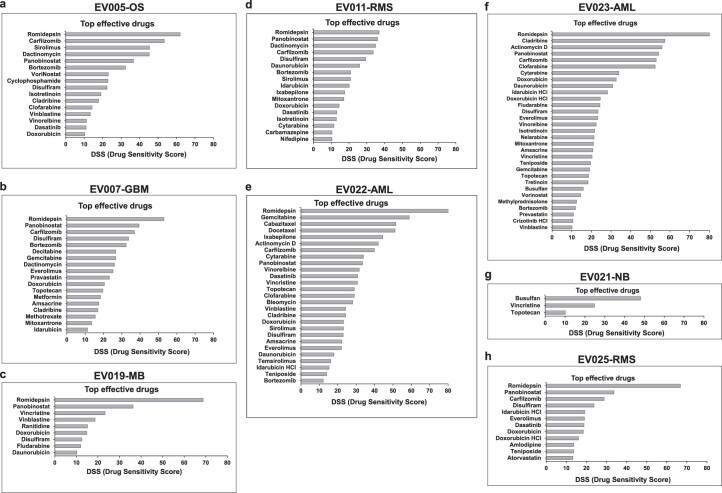
Top effective drugs for TPC patients. DSS for top effective agents, defined as DSS > 10, are shown for (**a**) EV005-OS, (**b**) EV007-GBM, (**c**) EV019-MB, (**d**) EV011-RMS, (**e**) EV022-AML, (**f**) EV023-ALL, (**g**) EV021-NB, and (**h**) EV025-RMS.

Notably, patients treated by TPC also had FPM data recommendations, reflected in the DSS waterfall plots (Extended Data Fig. 6); however, the treating physicians selected not to use the data to guide treatments for that cohort.

**Extended Data Fig. 7:**
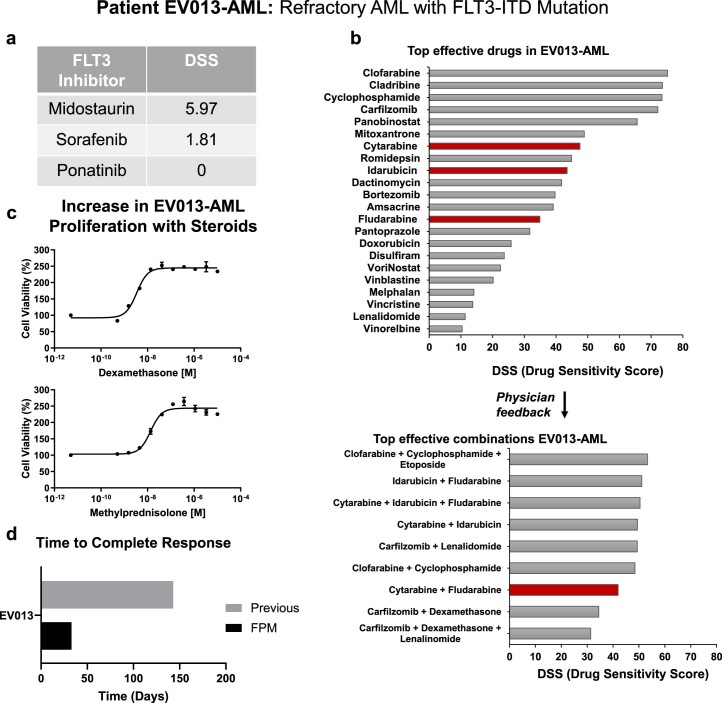
Additional data from EV013-AML. (**a**) DSS from clinically available FLT3 inhibitors (**b**) Top effective single agent drugs (top) followed by physician-selected drug combinations (bottom) (**c**) Dose-response from steroid agents tested in EV013-AML-derived cells. (n = 1 due to limited PDC material). Data is presented as mean cell viability values +/− SEM. (**d**) Comparison of time to complete response following previous and FPM-guided regimens.

Of particular interest is the case of an exceptional responder with AML (EV013-AML), who had treatment options identified through both genomics and drug testing. For this patient’s cancer, a clinically actionable *FLT3-ITD* mutation was identified, and DST was subsequently used to guide FLT3i selection. Testing revealed that midostaurin had the highest efficacy (DSS = 5.97) compared with sorafenib (DSS = 1.81) and ponatinib (DSS = 0), which demonstrated limited effectiveness (Extended Data Fig. 7a). DST data also indicated that fludarabine and cytarabine were effective enough without idarubicin, reducing toxicity for the patient (Extended Data Fig. 7b). Interestingly, DST results also identified acute proliferation of cells induced by steroids, which were subsequently withdrawn from the patient’s treatment plan (Extended Data Fig. 7c). These treatment decisions would not have been made without the FPM data, which led to both reduced time to complete response (33 days instead of 150 days with the previous treatment; Extended Data Fig. 7d) and increased durability of the second bone marrow transplant. This patient remains cancer free after more than 2 years; twice the PFS of the first bone marrow transplant. This case highlights the power of integrating DST with genomics to tailor treatments in real time for each patient.

### DST results correlate with clinical outcomes

To determine the predictive ability of our DST platform, we correlated DSSs of study treatments with clinical outcomes in 13 of the 14 patients who received therapeutic intervention during the study. Patient EV023-ALL, who received chimeric antigen reception T-cell therapy, was excluded, as this could not be tested by DST.

**Extended Data Fig. 8:**
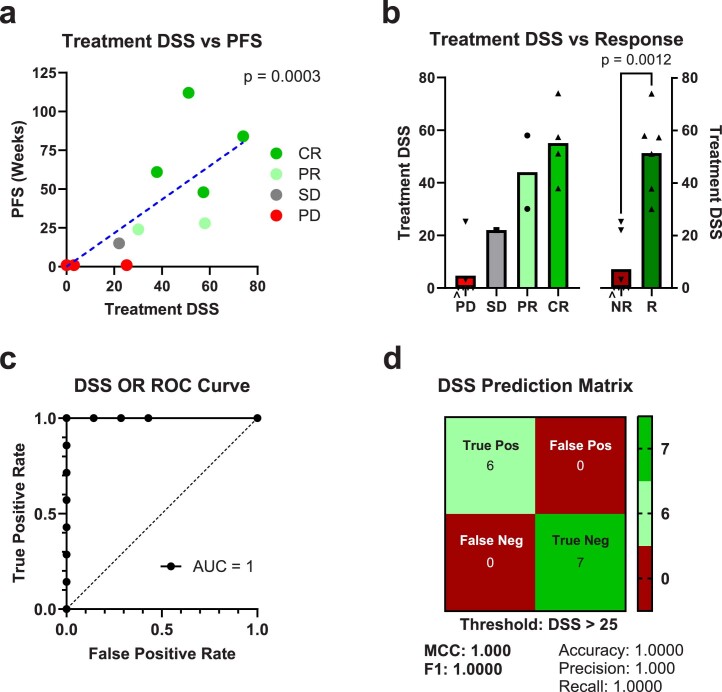
Post-hoc analyses correlating DST results with clinical outcomes. (**a**) Plot of the relationship between PFS and DSS of associated treatments in FPM-guided patients. P value is from two-sided Spearman correlation of DSS and PFS. Blue dashed line represents a line of simple linear regression. (**b**) Distribution of DSS separated by response type (left) and response class (NR = Non-Responder, R = Responder) in patients reviewed by the FPMTB. P value is from two-sided Mann-Whitney U test comparing DSS in R and NR classes. CR = complete response, PR = partial response, SD = stable disease, PD = progressive disease. Data are presented as mean values with individual points. In the left panel, PD represents n = 6 patients, SD represents n = 1 patient, PR represents n = 2 patients, CR represents n = 4 patients. In the right panel, NR represents n = 7 patients, R represents n = 6 patients. ^ indicates n = 4 points are at 0. (**c**) Receiver operating characteristic (ROC) curve of true positive rate and false positive rate of DSS-based response prediction. **d**) Confusion matrix and associated statistical values of DSS predicted and actual OR in FPM-guided patients and TPC patients at optimal threshold (DSS > 25). Prediction performance metrics (Accuracy, Precision/Positive Predictive Value, Negative Predictive Value, Recall, MCC, F1) are provided below the confusion matrix.

We identified a significant positive correlation between treatment DSS and PFS duration (*ρ* = 0.8732, *P* = 0.0003; Extended Data Fig. 8a and Supplementary Material – DST Correlation Data), suggesting that higher DSSs predict increased patient survival. We also identified a significant difference in study treatment DSS between cancers that responded (partial response or complete response) and non-responding cancers (stable disease or progressive disease) (*P* = 0.0012; Extended Data Fig. 8b), suggesting that higher DSSs correlate with improved ORRs. Furthermore, we used receiver operating characteristic (ROC) analysis to identify the optimal DSS cutoff to predict ORR (area under ROC curve = 1.000; Extended Data Fig. 8c). At the optimal DSS cutoff of DSS > 25, DST showed high predictive accuracy across all metrics (accuracy = 1.000, precision or positive predictive value = 1.000, negative predictive value = 1.000, recall = 1.000, Matthews correlation coefficient = 1.000, F1 test metric = 1.000) (Extended Data Fig. 8d).

**Extended Data Fig. 9:**
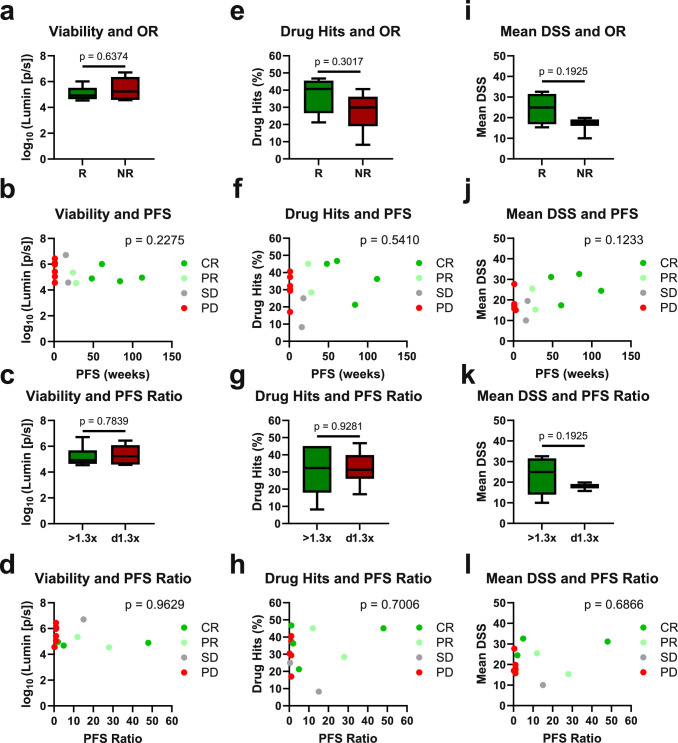
Post-hoc analyses correlating patient-specific clinical outcomes with DST assay measures. Analysis of relationship between viability of untreated control cells determined by luminescence, and (**a**) objective response (OR), (**b**) PFS, (**c**) PFS ratio ≥ 1.3x status, and (**d**) PFS ratio. Analysis of relationship between percentage of drugs showing any effectiveness and (**e**) OR (**f**) PFS, (**g**) PFS ratio ≥ 1.3x status, and (**h**) PFS ratio. Analysis of relationship between average DSS of drugs showing any effectiveness and (**i**) OR, (**j**) PFS, and (**k**) PFS ratio ≥ 1.3x status, and (**l**) PFS ratio. No significant relationship was identified between any confounding variable and any outcome measure. P values in a, c, e, g, l, and k determined by two-sided Kolmogorov-Smirnov tests comparing medians of classes. P values in b, d, f, h, j, and l determined by two-sided Spearman correlation analyses comparing confounding variables with outcomes. R = Responder, NR = Non-Responder, CR = complete response, PR = partial response, SD = stable disease, and PD = progressive disease. In all box and whisker plots in panels a, c, e, g, i, and k, the lower box line represents the low quartile (25th percentile), the center line represents the median (50th percentile), the top line represents the upper quartile, and the whiskers represent the minimum and maximum. R represents n = 6 patients, and NR represents n = 8 patients. Similarly, ≥1.3x represents n = 6 patients, and <1.3x represents n = 8 patients.

We also performed post-hoc analysis correlating patient-specific clinical outcomes with DST assay measures including viability measures in untreated control cells, number of drug hits (percentage of drugs with DSS > 0) and average DSS among all drugs with any effectiveness. No significant relationships were identified among any of the three DST measures (*P* > 0.05 for all comparisons; Extended Data Fig. 9 and Supplementary Table – Assay correlation data), suggesting that clinical outcome improvement is not attributed to confounding patient-specific characteristics, and instead can be attributed to interventions provided during the study.

Taken together, these analyses demonstrate that DST data are a strong predictor of clinical response and DST guidance can improve clinical outcomes, independent of confounding clinical factors. These findings further emphasize the potential of DST as a valuable tool for guiding treatment decisions in high-risk malignancies, including pediatric and adolescent cancers.

## Discussion

We demonstrate the feasibility of returning a combination of drug sensitivity profiles and molecular data (FPM) to clinicians to inform subsequent treatment recommendations for pediatric patients with relapsed or refractory cancers. This prospective study highlights the use of FPM data to inform the next line of therapy for children who have exhausted standard-of-care options. We provided actionable treatment options for 84% of enrolled patients. DST results were available within a median of 9 and 10 days for hematological and solid tumors, respectively, giving the physicians treatment recommendations in a clinically relevant timeframe. Those treatments were later modified with a targeted drug if an actionable genomic mutation was found. Additionally, we demonstrate that 83% of patients who received FPM-guided treatment had an improved best overall response (partial response or better) and a median 8.5-fold increase in PFS compared to their previous regimens. Conversely, 13% of patients receiving TPC achieved an objective response, consistent with anticipated outcomes for hard-to-treat refractory pediatric and adolescent cancers previously treated with multiple lines of therapy^32^ and emphasizing the need for more refined treatment options.

Results from the INFORM registry study suggest that patients who did not receive matched treatments had a median PFS of 16.2 weeks (3.8 months) across all cancer types; notably, this study enrolled patients across all clinical stages and as early as at first diagnosis^10^. Although direct comparisons of outcomes are challenging in advanced refractory childhood cancers, we found improved tumor-specific outcomes in our study compared to the INFORM registry (Supplementary Table; see Expected PFS).

Other recent studies demonstrating the feasibility of FPM have focused on adult patients with leukemia and lymphoma^14,15^. Studies such as INFORM in Europe have started to investigate the potential clinical utility of integrating DST to their genomic platforms^9^; however, to our knowledge, no prospective FPM studies in children have been performed. Our prospective study includes both liquid and solid tumors, regardless of cancer type, thus demonstrating broader application of FPM and expanding access to refined personalized treatment options. Furthermore, targeting pediatric and adolescent cancer addresses a critical gap in current treatments.

As the primary objective was to assess the feasibility of delivering FPM data to the clinic, a relatively small cohort was followed and did not include a randomized control group. In addition, as we included both liquid and solid tumors in our study, we did not collect extensive outcome data for any particular cancer type owing to cancer type heterogeneity, limiting our ability to compare outcomes statistically within one tumor type. To evaluate the effect of FPM in guiding therapy across heterogeneous diseases and disparate treatment regimens, we instead reviewed patients’ PFS ratios, a common approach in precision medicine trials in which each patient’s clinical outcome serves as its own control^14,15,26,33^.

We also acknowledge that our patients’ experiences with previous treatments may have limited tumor response to new therapies and that rapid disease progression experienced by some patients in our study may have limited the implementation of guided treatment options. Although turnaround time can be further reduced, the median turnaround time for DST testing of 9–10 days spotlights the dire challenges faced by patients with severely advanced disease, suggesting the need for earlier implementation of guided approaches to better assess clinical utility. Despite these limitations, our results suggest that a broad range of chemotherapeutic drugs and targeted inhibitors are capable of overcoming drug resistance, even in heavily refractory cancers.

Recent precision medicine studies have reported the significant barriers to targeted treatment for their patients, including deteriorating disease, access to off-label use, financial restrictions and—in the case of pediatric patients—limited dosage guidelines and efficacy data in children^5,7^. In our study, these hurdles often resulted in the clinicians relying on the FPM recommendations of more readily accessible drugs, as they often encountered resistance to off-label use of more targeted treatments with high ex vivo efficacy such as histone deacetylase inhibitors and proteosome inhibitors. Overcoming these obstacles to targeted oncology drugs will require collaboration between regulatory bodies, researchers, pharmaceutical companies, and patient advocacy groups to advance both genomics-guided and FPM-guided medicine. This study also emphasized, as have other precision oncology studies, that patient access to guided treatments may depend on physicians’ attitudes towards emerging technologies and methodologies. Throughout the course of the study, we learned that physician acceptance of FPM-guided recommendations was an important endpoint that had not been considered. The acceptance and impact of FPM programs will thus depend on physician education, and increasing familiarity with new approaches in oncology and new types of data that will influence clinical decision-making. Therefore, current and future clinical trials should assess acceptance as an exploratory endpoint.

One limitation of DST studies, as suggested in the TUMOROID study^34^, is that some treatments may rely on immune and/or stromal cells present in the tumor environment, which may not be fully recapitulated in culture models derived solely from the epithelial compartment^35,36^. Therefore, our culture models, which are mixed cell populations that include immune cells, may more adequately represent the tumor.

Another challenge is the heterogeneity between primary and metastatic lesions^37^, which leads to variation in drug sensitivity and requires concurrent evaluation of both sites to predict efficacious therapeutic regimens. Recognizing this limitation, our currently enrolling pediatric study (ClinicalTrials.gov registration: NCT05857969) procures both primary and metastatic lesions whenever possible.

Overall, the addition of functional drug testing to current personalized medicine platforms has promising potential to expand treatment options when limited alternatives exist. This is especially valuable when assessing drugs whose mechanisms of action are poorly understood or not robustly characterized. Moreover, our ability to screen multiple monotherapy and combination therapy options with high clinical accuracy, and to provide drug response data within a clinically actionable timeframe, supports the feasibility and efficacy of FPM approaches, indicating the need for continued validation to make these approaches accessible for the treatment of rare and high-risk cancers. The observed improvement in objective response and increase in overall PFS, especially compared to patients’ previous treatment results, highlights the importance of moving closer to clinical integration of functional DST with existing genomic profiling to improve patient outcomes. Nevertheless, our clinical cohort was small and heterogeneous with respect to tumor type, which represents an important limitation of this study. At this stage, conclusions drawn are preliminary and require further validation. Accordingly, we are continuing our validations efforts with larger clinical studies, including our actively enrolling studies for patients with childhood cancer (ClinicalTrials.gov registration: NCT05857969) and adult cancer (ClinicalTrials.gov registration: NCT06024603).

**Extended Data Fig. 10:**
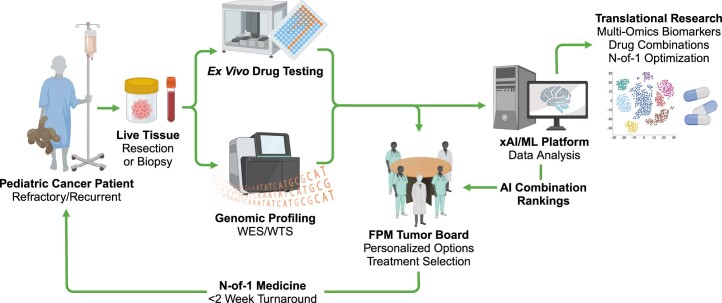
Integration of FPM and explainable artificial intelligence/machine learning (xAI/ML) for advancing personalized medicine workflows. Workflow diagram depicting the sequential process of the FPM and xAI/ML approach for enhancing individualized cancer medicine. Patients are enrolled followed by a biopsy/resection of the tumor sample. Live patient-derived cultures undergo high-throughput ex vivo DST assay in combination with molecular tumor profiling using whole-exome sequencing and whole-transcriptome sequencing. The results of both the DST and molecular profiling are reported to the FPM tumor board (FPMTB) to make informed treatment decisions based on each individual patient’s profile. The xAI/ML platform simultaneously analyzes DST results, molecular profiling data and existing knowledge of drug interactions to provide potential drug combinations tailored to each patient’s specific tumor characteristics, as well as uncovers potential multi-omics biomarkers. The drug combination rankings will also be reported to the FPM tumor board for treatment decision-making. The process will enable the FPMTB to make treatment decisions in a clinically actionable timeframe (less than 2 weeks) for each individual patient. The workflow shows the multidimensional and personalized approach for further development of personalized cancer medicine. Created with BioRender.com.

Last, as FPM approaches become increasingly adopted in clinical practice, and the availability of paired functional and molecular datasets grows, we anticipate the development of a future collaborative workflow that incorporates artificial intelligence and machine learning technologies into FPM^38–40^. This integrated approach will incorporate functional drug response data with molecular profiling and pathway information, serving as the foundation for refining individualized treatments, advancing FPM strategies, and identifying novel predictive biomarkers (Extended Data Fig. 10).

## Methods

### Study design

Our feasibility study enrolled patients from 21 February 2019 to 31 December 2022 (ClinicalTrials.gov registration: NCT03860376). All patients provided written informed consent at the time of enrollment to participate in the study, including consent to publish, and the study was approved by the Western Institutional Review Board and Ethics Committee (IRB no. 1186919). Patients of any gender, race or ethnicity were eligible for inclusion in the study if they met the following inclusion criteria: they were aged 21 years or younger at the time of enrollment; had suspected or confirmed diagnosis of recurrent or refractory cancer; were scheduled for or had recently had biopsy or tumor excision (solid tumors) or bone marrow aspiration (blood cancers); were willing to have a blood draw or buccal swab done for the purposes of genetic testing; they or their parents or legal guardians were willing to sign informed consent; and, for patients aged 7 to 17, they were willing to sign assent. Patients’ biological sex and ethnicity were recorded based on self-reporting.

Patients were excluded based on the following exclusion criteria: if they did not have malignant tissue available and accessible; if the amount of excised malignant tissue was not sufficient for ex vivo drug testing and/or genetic profiling; and if they had a newly diagnosed tumor or a tumor with a high (>90%) cure rate with safe standard therapy. The primary outcome was return of actionable treatment recommendation(s) from FPM data, consisting of DST and/or genomics data, within a clinically actionable time frame (within 4 weeks). The primary endpoint of this study was the percentage of patients receiving treatment options through FPM data within a 4-week timeframe, with a null hypothesis of <30% of patients meeting the promary endpoint. The objective would be considered met, and the null hypothesis rejected, if treatment options were returned to at least 60% of enrolled patients. At initiation of the study, we anticipated enrolling 16 patients, and determined that successfully returning clinically actionable treatment options through FPM to 10 patients (62.5% of enrolled patients) would provide 80% power to reject the null hypothesis (90% CI 0.492–1). After initiation of the study, our budget expanded, allowing us to enroll additional patients and increase statistical power at a similar target success rate. The secondary objectives included in the study reflect those that are now commonly reported in genomics and FPM studies^15,26–29^, including ORR between cohorts, PFS between cohorts and PFS2/PFS1 ratio metrics between the study regimen and the most recent previous regimen of the same patient above a defined threshold (1.3×). Note that the PFS2/PFS1 ratio metric was added to the amended statistical analysis plan after trial initiation owing to this metric becoming routinely used in precision cancer medicine studies. Exploratory analyses interrogated the correlation between DSS values from DST assays and clinical outcomes, as well as relationships between disease aggressiveness and responsiveness metrics from DST assays and clinical outcomes.

### Tumor processing and PDCs

Tumor samples were collected from 24 out of 25 enrolled pediatric and adolescent patients with relapsed or refractory solid or hematological cancers. All primary tumor samples were collected fresh and sent to our laboratory for processing within 24–48 h.

The same tissue processing protocol was used for all solid tumor tissue samples, as previously described^12,41^. In brief, solid tissue samples from enrolled patients were mechanically dissociated using scalpels before being enzymatically dissociated with both DNase I (Invitrogen) and Liberase DH (Roche) for approximately 90 min at 37 °C (Fig. 2). Red blood cells were subsequently removed from dissociated samples through lysis with ACK Lysing Buffer (Gibco), then cells were cultured overnight in RPMI 1640 medium supplemented with antibiotics (100 U ml^−1^ penicillin and 100 μg ml^−1^ streptomycin) and 10% FBS (Gibco). Cells were seeded for drug screening following an appropriate culturing period, determined by the morphological characteristics and growth dynamics of the PDCs; drug screening for most samples occurred 1–3 days following tumor dissociation. Any adherent cells from solid tumor PDCs were detached using TrypLE Express (Gibco) before drug screening. Mononuclear cells were isolated from hematological cancer samples using SepMate PBMC Isolation Tubes (STEMCELL Technologies) and Ficoll-Paque PLUS density gradient (Cytiva) according to the manufacturers’ instructions, as previously described^13,18^, and cultured in Mononuclear Cell Medium with Supplement (PromoCell). All PDCs were closely monitored by light microscopy following tumor dissociation and were cultured a minimum of 12 h before proceeding with DST.

### DST

Cells from PDCs were seeded into white 384-well microplates (Thermo Fisher Scientific) with 1,000 cells per well. The following day, drugs were added at appropriate concentrations using an epMotion P5073 liquid handler (Eppendorf). A custom drug library (ApexBio) was used for DST, encompassing formulary drugs from Nicklaus Children’s Hospital, non-formulary FDA-approved cancer drugs and phase III or IV oncology drugs and additional non-cancer agents that have been investigated for potential repurposing as anticancer treatments. Drugs were tested in duplicate at ten concentrations from 10 μM to 0.5 nM (ref. ^12^), along with DMSO (negative control) and 100 μM benzethonium chloride (positive control). Several patient samples were tested with additional drugs at the request of the treating physician. Additionally, samples from three patients (EV021-NB, EV024-ALL, EV025-RMS) underwent partial library testing owing to small sample size. All cells were subsequently incubated at 37 °C and 5% CO_2_ for 72 h. Cell viability was then assessed by evaluating cellular ATP using CellTiter-Glo (for hematological cancers) or CellTiter-Glo 3D (for solid tumors) luminescent cell viability assay (Promega) according to the manufacturer’s protocol. Luminescence was measured using a multimode plate reader (Perkin Elmer). The resulting luminescence data were used to generate dose–response curves to derive DSSs using GraphPad Prism 8 and the DSS v.1.2 package in R v.3.6.3, as previously described^12,18,20^.

### Quality control analysis of DST assays

Following assay endpoint readout through CellTiter-Glo or CellTiter-Glo 3D, raw luminescence data from negative control wells (DMSO) and positive control wells (benzethonium chloride) were used to generate per-plate *Z*-prime scores^19^. In brief, the *Z*-prime score uses the mean and s.d. of positive and negative controls within a single assay plate to determine assay quality. The *Z*-prime score is defined with the following relationship:

$$1-\frac{3\left({\sigma }_{p}+{\sigma }_{n}\right)}{\left|{\mu }_{p}-{\mu }_{n}\right|}$$

where *µ*_*p*_, *σ*_*p*_ and *µ*_*n*_, *σ*_*n*_ are the sample mean and s.d. for the positive and negative control, respectively. Assays with *Z*-prime scores in the range 0.5–1 were considered high-quality assays, those in the range −0.5–0.5 were considered marginal quality assays, and those below −0.5 were considered failed assays. High-quality assays and marginal assays with median luminescence values of >5,000 passed quality control, all other assays failed quality control (Fig. 2b). Our quality control process was adapted from previous high-throughput screening quality control approaches^9^.

### Genomic panel sequencing

For solid cancers, formalin-fixed paraffin-embedded (FFPE)-preserved tissue sections from surgical samples and matched whole blood from patients were sent to the UCSF Clinical Cancer Genomics Laboratory for UCSF500 Cancer Gene Panel sequencing. For hematological cancers, whole blood and patient-matched buccal swabs were sent instead. Samples from all patients enrolled in the study underwent genomic tumor profiling, provided sufficient tissue was available. In addition, several patients underwent genomic panel sequencing services through Foundation Medicine or CHLA OncoKids before involvement in the study; we report results from these sequencing services, when available. Analyte isolation, physical sequencing and clinical interpretation were performed by each respective service.

### Pediatric and adolescent FPMTB

Results from DST and genomic panel sequencing for each patient were made available as soon as possible to the FPMTB, which comprised treating physicians (*n* = 4), pharmacists (*n* = 2), hematology or oncology nurses (*n* = 3), precision medicine specialists (*n* = 1) and clinical research coordinators (*n* = 2) from Nicklaus Children’s Hospital, as well as translational researchers (*n* = 3) from Florida International University. Upon receiving results, the FPMTB convened to evaluate the data, consider the availability for off-label use of candidate drugs and review the treatment histories of each patient. Subsequently, a final list of therapeutic options, ranked in order of preference along with recommended doses and schedules, was provided for each patient^12^. The board also carried out follow-up analysis of treatment responses for eligible patients. More details for patients, treatment selection and outcomes are shown in the Supplementary Table (see Clinical outcomes).

### Immunofluorescence analysis of PDCs

Tumor-derived cultures from patients EV004-RMS, EV010-EWS and EV019-MB were assessed by immunofluorescence for the presence of markers described in the surgical pathology reports, namely desmin, myogenin, NKX2.2 and beta-catenin, respectively. Cells were fixed with 4% PFA for 10 min at room temperature (20–22 °C) before blocking and permeabilization was performed with a HBSS-based solution containing 5% normal bovine serum albumin (Thermo Fisher Scientific), 0.2% Tween-20 (Thermo Fisher Scientific) and 0.1% Triton X-100 (Thermo Fisher Scientific). Cells were incubated overnight with the primary antibodies, either anti-Desmin (D93F5) (Cell Signaling), anti-MyoD1 (D8G3) (Cell Signaling), anti-NKX2.2 (EPR14638) (Abcam) or anti-β-Catenin (D10A8) (Cell Signaling), at 4 °C and then washed with HBSS three times. Cells were then incubated with Alexa Fluor secondary antibodies (Life Technologies, 1:500 dilution) for 1 h before undergoing another series of washes following secondary antibody incubation. Cells were mounted on slides and cover slipped with Prolong Gold Antifade Mountant (Life Technologies) to preserve signal intensity and brightness. Labeled cells were imaged using a laser scanning confocal microscope (Fluoview FV10-ASW v.04.02.02.09, Olympus), using the FV10-AWS v.04.02.02.09 image software. Owing to limited material in the PDCs, immunofluorescence experiments were performed once for each patient, with technical replicates and appropriate controls.

### RT–qPCR analysis of gene deletions in PDCs

Gene expression for *TP53* and *DIS3L2* was assessed from RNA isolated from cultures derived from EV003-OS and EV015-WT tumor samples, respectively, as well as normal human skeletal muscle cells. RNA was isolated using the RNeasy Mini Kit (Qiagen), and concentration was measured with a NanoDrop One spectrophotometer (Thermo Fisher Scientific). RT–qPCR was performed in a QuantStudio 6 Flex (Life Technologies) using TaqMan Fast Advanced Master Mix and primers for *TP53* (Hs01034249_m1 FAM-MGB, Thermo Fisher Scientific, no. 4331182), *DIS3L2* (Hs04966835_m1 FAM-MGB, Thermo Fisher Scientific, no. 4351372) and GAPDH (Hs02758991_g1 VIC-MGB, Thermo Fisher Scientific, no. 4331182). Results were evaluated using QuantStudio Real-Time PCR System Software v.1.3 (Thermo Fisher Scientific). Amplification specificity was confirmed by melting curve analysis, and quantification was performed using ΔΔCt (ref. ^42^). All samples were normalized to GAPDH and compared with normal human skeletal muscle cells.

### Whole exome and whole transcriptome sequencing and analysis

DNA and RNA isolation for solid tumor samples was performed from sectioned FFPE tissue stored at Nicklaus Children’s Hospital. Tissue sectioning was performed by HistoWiz. The Beijing Genomics Institute (BGI) performed analyte isolation from FFPE curls. FFPE tissues were shipped to BGI at ambient temperatures separately from DNA and RNA.

DNA and RNA isolation for sequencing of hematological cancer samples was performed using Qiagen DNA and RNA Mini-Prep kits according to the manufacturer’s instructions. Cryopreserved PDC samples derived from solid cancer samples were shipped overnight on dry ice for DNA and RNA isolation by BGI or Novogene for subsequent sequencing. Frozen isolated DNA and RNA were shipped overnight on dry ice for physical sequencing by BGI or Novogene.

Sequencing was performed by BGI using the DNBSeq G400 sequencer and by Novogene using the Illumina NovaSeq6000 sequencer, and data were analyzed using previously established analysis pipelines based on best practices^39,40,43,44^. In brief, raw FASTQ sequencing files from DNA sequencing experiments were quality control-filtered using SOAPnuke v.2.1.8 (ref. ^45^) and aligned to the GRCh38 human reference genome using BWA MEM aligner in the BWA v.0.7.17 package^46^. Somatic mutations and indels were called using Genome Analysis Toolkit (GATK) v.4.0 according to best practices for tumor-only samples^47–49^.

Post-quality-control RNA sequencing data were aligned to the reference transcriptome using the STAR v.2.7.10b aligner^50^, gene expression was quantified using RSEM v.1.3.3 (ref. ^51^), and gene fusion events were detected using STAR-Fusion v.1.9 (ref. ^52^). To call variants from RNA sequencing data, post-quality-control FASTQ files were aligned to the GRCh38 human reference genome using STAR v.2.7.10b and processed using GATK v.4.0 following best practices for RNA-seq short variant discovery to identify somatic mutations and indels present in the transcriptome.

The list of all next-generation sequencing experiments performed is provided in the Supplementary Table (see NGS samples).

### RNA-seq analysis and tumor purity of PDCs

Post-processing gene expression data from RNA sequencing analysis were analyzed for cell population content focusing on stromal and/or immune cell populations. Four separate tools were used to perform cell population analysis: (1) ESTIMATE^53^ analysis performed using the tidyestimate v.1.1.1 package in R v.4.3.0; (2) quanTIseq^54^ analysis performed in R v.4.3.0 through Singularity v.3.8.7; (3) TIMER2.0 (ref. ^55^) analysis performed through the TIMER2.0 web portal (http://timer.cistrome.org); and (4) EPIC^56^ analysis performed in R v.4.3.0 using the EPIC v.1.1.7 package. All four tools deconvolute gene expression data using prebuilt signatures for immune and/or stromal cell populations. Graphs from analysis results were prepared in Prism 10.0, and RNA deconvolution data are provided in the Supplementary Table (see RNA deconvolution).

### Statistics and reproducibility

Hypothesis testing for differences in PFS between FPM-guided and TPC cohorts was performed using a two-sample logrank (Mantel–Cox) test. Hypothesis testing for differences in PFS between previous and current regimens (in both FPM-guided and TPC cohorts) was performed using Cox regression with clustered computation, owing to the intracohort analysis representing repeated measures. Hypothesis testing for changes in PFS ratio between the previous regimen and the trial regimen (in both FPM-guided and TPC cohorts) was performed using the Wilcoxon matched pairs test. Hypothesis testing for differences in the incidence of a PFS ratio of ≥1.3× between the previous regimen and the trial regimen (in both FPM-guided and TPC cohorts) was performed using Barnard’s test. Kaplan–Meier curve generation and analysis were performed in GraphPad Prism 10.0. Barnard’s unconditional test of superiority was performed using the Barnard v.1.8 package in R v.3.6.3. The exact binomial test was performed in R v.3.6.3. Cox regression with clustered computation was performed in R v.3.6.3 using the ‘coxph’ function in the survival v.3.1-8 package. Mann–Whitney *U*-tests, Kolmogorov–Smirnov tests, McNemar’s test with continuity correction, Kruskal–Wallis tests, Chi-square tests, Spearman correlation coefficient analysis and Wilcoxon matched pairs tests were performed in GraphPad Prism 10.0. Except for the one-sided exact binomial test used to analyze the primary outcome measure, all statistical tests performed are two-sided, where appropriate. Statistical tests, uses, results, sidedness and software packages are further described in the Supplementary Table (see Statistical tests and tools). The statistical analysis plan is included in the Supplementary Information.

Owing to the limited sample available from each patient and the requirement to return results in a clinically relevant timeframe, ex vivo DST was performed as *n* = 1 biological replicate for each patient. Technical replicates and positive and negative controls for DST were included on each plate. The tissue limitations also affected the number of experiments that were performed for PDC validation studies, including genomic and transcriptomic analysis (*n* = 1 biological replicate) and immunofluorescence analysis (*n* = 1 biological replicate). However, multiple validation approaches were used on the same sample, affirming the biological relevance of our PDC models (*n* = 11 independent biological samples).

### Reporting summary

Further information on research design is available in the Nature Portfolio Reporting Summary linked to this article.

## Online content

Any methods, additional references, Nature Portfolio reporting summaries, source data, extended data, supplementary information, acknowledgements, peer review information; details of author contributions and competing interests; and statements of data and code availability are available at 10.1038/s41591-024-02848-4.

## Supplementary information

Supplementary Information Table of contents for Supplementary Tables excel file Statistical Analysis Plan

Reporting Summary

Supplementary Table All supplementary tables included in the manuscript. The table of contents is included as a PDF and as the first sheet in the Supplementary Tables file.

## Data Availability

All materials generated during our study and used in our analysis are provided in the tables or supplementary tables. The GRCh38 human reference genome is available through Ensembl (https://ftp.ensembl.org/pub/release-111/fasta/homo_sapiens/dna). The GRCh38 gencode v.22 CTAT transcriptome library is available through the Broad Institute (https://data.broadinstitute.org/Trinity/CTAT_RESOURCE_LIB). Raw sequencing data are available through the European Genome-Phenome Archive (EGA) under accession number EGA50000000164.

## Code availability

No custom code was generated during this study.
