# Supplementary material for: Feasibility of functional precision medicine for guiding treatment of relapsed or refractory pediatric cancers
Source: Nat Med. 2024 Apr 11;30(4):990–1000. doi: 10.1038/s41591-024-02848-4 (PMC11031400; doi:10.1038/s41591-024-02848-4)
Supplement: Supplementary file 1 — Table of contents for Supplementary Tables excel file Statistical Analysis Plan [file 41591_2024_2848_MOESM1_ESM.pdf]

nature medicine

Article

https://doi.org/10.1038/s41591-024-02848-4

Feasibility of functional precision medicine for guiding treatment of relapsed or refractory pediatric cancers

In the format provided by the authors and unedited

Supplementary Tables – Table of Contents

- Testing and Demographics
    - Patient characteristics, demographics, clinically relevant information, and outcomes from diagnostic testing
- Drug List
    - List of drugs included in drug testing panel and associated drug mechanisms
- Culture Validation Experiments
    - Validation tests performed on patient-derived cultures and associated results
- Actionable Panel Seq Results
    - Actionable or potentially actionable variants identified from tumor panel sequencing tests
- Complete Panel Seq Results
    - Results from all tumor panel sequencing tests performed on patient samples during the study
- DST Testing Results
    - DSS score for each drug tested on every patient sample during DST
- Z' Statistics
    - Z' Statistic Scores from individual assay plates and associated plates that failed QC
- DST Repeat Data
    - DSS and IC50 values for set of repeated DST experiments performed during the course of the study
- DST Combination Results
    - DSS values from physician-directed combination DST experiments
- DST Correlation Data
    - Data relating response type and progression-free survivial with associated treatment DSS values
- Assay Correlation Data
    - Quantified assay measures (Hit %, Avg DSS, raw cell viability from CTG) and associated clinical outcomes
- Expected PFS
    - Expected PFS of individual pediatric cancer types curated from recent INFORM genomics study
- Clinical Outcomes
    - Patient inclusion/exclusion critera and trial stage, objective response, and associated progression-free survival for all patients
- NGS Samples
    - Samples and approaches used for NGS
- RNA Deconvolution
    - Results from deconvolution tool analysis of bulk RNA-seq data
- Statistical Tests and Tools
    - List of specific statistical tests performed for data analysis in this study

Statistical Analysis Plan

Descriptive statistics will be used to assess (categorical count frequency and percentage)

- Age of patients at enrollment
- Ethnicity of patients
- Sex of patients
- Incidences of specific cancer types, including supertypes (e.g. leukemia is a heme malignancy, rhabdomyosarcoma is a sarcoma)
- The number of enrolled patients providing tissue samples
- The number of patients receiving drug sensitivity testing data
- The number of patients receiving genomic tumor profiling data
- Turnaround time from tissue provision to return of drug sensitivity testing data
- Turnaround time from tissue provision to return of genomics testing data
- Actionability of drug sensitivity testing data, defined as identifying a clinically-usable treatment recommendation from testing
- Actionability of genomics testing data, defined as identifying a clinically-usable treatment recommendation from testing
- Trial progression status

Data underlying descriptive stats are available in the Supplementary Tables.

The primary endpoint of this study is patients receiving clinically-actionable treatment recommendations through Functional Precision Medicine, defined as drug sensitivity testing (DST) data and/or genomics data in a clinically-actionable time frame (within 4 weeks), with a null hypothesis of <30% of patients receiving FPM data and meeting the endpoint.

- To test this hypothesis, a one-sided exact binomial test will be applied with an alpha level of 0.025. To achieve at least 90% power, the null hypothesis will be rejected when at least 16 out of 25 patients receive FPM data within 4 weeks on the study. With that outcome, we would have 95% confidence that the true feasibility rate is at least 30% (95% CI: 0.425, 1).

At conclusion of the study

- 24 of 25 patients provided tumor tissue samples
- 21 of 25 received drug sensitivity testing data
- 20 of 25 received genomics testing data
- 19 of 25 patients received both drug sensitivity testing data and genomics testing data
- One-sided exact binomial analysis of the resulting FPM data delivery demonstrates the true data delivery rate is significantly greater than 30% (19 of 25 enrolled patients (76%), p = 2.767e-06, 95% confidence interval = [0.5487 to 0.9064]). Analysis of DST data alone demonstrates the true delivery rate is also significantly greater than 30% (21 of 25 enrolled patients (84%), p = 3.439e-08, confidence interval = [0.6704 to 1.0000]), which is identical to the actionability rate of DST data (21 of 25 provided samples, p = 3.439e-08, confidence interval = [0.6704 to 1.0000]).

The secondary endpoints of the study compare clinical impact of therapy selection through the use of FPM or through non-FPM guided (conventional) therapy. To address this goal, we will apply hypothesis testing to multiple clinically-relevant endpoints.

Objective Response Rate

- Objective Response Rate (the percentage of responders among total evaluable patients) in the FPM guided cohort vs the conventional protocol cohort will be calculated. A responder to the treatment is defined as any patient who achieves the best response of "Partial Response" or "Complete Response" during the study period, with these response types determined by the individual physicians per standard guidelines for both solid cancers and hematological cancers.
- Comparisons of the Objective Response to previous treatment and trial treatments (FPM-guided prior vs FPM-guided trial and conventional prior vs conventional trial) will be calculated using a two-sided McNemar's test for paired binary data with continuity correction.
- Comparison of Objective Response Rate during the trial between FPM-guided and conventional cohorts will be performed using Barnard's test.
- Distribution of Objective Responses and associated statistical results are in the Supplementary Tables

Progression-Free Survival

- Hypothesis testing for differences in Progression-Free Survival (PFS) between FPM-guided and conventional therapy will be performed using a two-sample log-rank (Mantel-Cox) test.
- Hypothesis testing for differences in PFS between previous and trial regimens in both FPM-guided and conventional cohorts will be performed using Cox regression with clustered computation, due to the intracohort analysis representing repeated measures.
- Hypotheses testing for differences in Progression-Free Survival ratio between previous regimen and trial regimen (in both FPM-guided and conventional cohorts) will be performed using Wilcoxon matched pairs test.
- Previous and trial PFS values for each patient by treatment cohort and associated statistical results are provided in the Supplementary Tables
- Previous vs trial PFS for the conventional arm and the FPM-Guided arm are in the Supplementary Tables

Previous vs. Trial Progression-Free Survival Ratio

- Hypotheses testing for differences in incidence of Progression-Free Survival ratio ≥ 1.3x between previous regimen and trial regimen (in both FPM-guided and conventional cohorts) will be performed using Barnard's test.
- Paired previous vs trial PFS values and associated statistical results are available in the Supplementary Tables
