# Supplementary material for: Feasibility of functional precision medicine for guiding treatment of relapsed or refractory pediatric cancers
Source: Nat Med. 2024 Apr 11;30(4):990–1000. doi: 10.1038/s41591-024-02848-4 (PMC11031400; doi:10.1038/s41591-024-02848-4)
Supplement: Supplementary file 2 — Reporting Summary [file 41591_2024_2848_MOESM2_ESM.pdf]

nature portfolio

Corresponding author(s): Diana J. Azzam

Last updated by author(s): Jan 31, 2024

Reporting Summary

Nature Portfolio wishes to improve the reproducibility of the work that we publish. This form provides structure for consistency and transparency in reporting. For further information on Nature Portfolio policies, see our Editorial Policies and the Editorial Policy Checklist.

Statistics

For all statistical analyses, confirm that the following items are present in the figure legend, table legend, main text, or Methods section.

n/a	Confirmed	
☐	☒	The exact sample size (*n*) for each experimental group/condition, given as a discrete number and unit of measurement
☐	☒	A statement on whether measurements were taken from distinct samples or whether the same sample was measured repeatedly
☐	☒	The statistical test(s) used AND whether they are one- or two-sided<br>*Only common tests should be described solely by name; describe more complex techniques in the Methods section.*
☐	☒	A description of all covariates tested
☐	☒	A description of any assumptions or corrections, such as tests of normality and adjustment for multiple comparisons
☐	☒	A full description of the statistical parameters including central tendency (e.g. means) or other basic estimates (e.g. regression coefficient) AND variation (e.g. standard deviation) or associated estimates of uncertainty (e.g. confidence intervals)
☐	☒	For null hypothesis testing, the test statistic (e.g. *F*, *t*, *r*) with confidence intervals, effect sizes, degrees of freedom and *P* value noted<br>*Give P values as exact values whenever suitable.*
☒	☐	For Bayesian analysis, information on the choice of priors and Markov chain Monte Carlo settings
☐	☒	For hierarchical and complex designs, identification of the appropriate level for tests and full reporting of outcomes
☐	☒	Estimates of effect sizes (e.g. Cohen's *d*, Pearson's *r*), indicating how they were calculated

Our web collection on statistics for biologists contains articles on many of the points above.

Software and code

Policy information about availability of computer code

Data collection	No special software was used for data collection in this study
Data analysis	Immunofluorescence was captured using the FV10-ASW version 04.02.02.09 image software.<br><br>GraphPad Prism v8 was used during the course of the study for dose-response curve fitting.<br><br>GraphPad v10 was used for post-hoc data analysis.<br>R v3.6.3 and the following packages were used in data analysis during this study: DSS v1.2, Barnard v1.8 package, survival v3.1-8.<br>The following packages were used in R v4.3: EPIC v1.1.7, tidyestimate v1.1.1, quanTIseq v1.1 package via Singularity v3.8.7.<br>TIMER2.0 analysis was performed through the TIMER2.0 web portal (http://timer.cistrome.org/).<br>qRT-PCR results were evaluated using QuantStudio Real-Time PCR Software v1.3.<br><br>The following software packages were used for analysis of genomics and transcriptomics data:<br>SOAPnuke v2.1.8, BWA v0.7.17, Genome Analysis Toolkit (GATK) Version 4.0, STAR v2.7.10b, STAR-Fusion v1.9, RSEM v1.3.3

For manuscripts utilizing custom algorithms or software that are central to the research but not yet described in published literature, software must be made available to editors and reviewers. We strongly encourage code deposition in a community repository (e.g. GitHub). See the Nature Portfolio guidelines for submitting code & software for further information.

Data

Policy information about availability of data

All manuscripts must include a data availability statement. This statement should provide the following information, where applicable:

- Accession codes, unique identifiers, or web links for publicly available datasets
- A description of any restrictions on data availability
- For clinical datasets or third party data, please ensure that the statement adheres to our policy

All materials generated during our study and used in our analysis are provided in main or supplementary tables. The GRCh38 human reference genome is available through Ensembl (https://ftp.ensembl.org/pub/release-111/fasta/homo_sapiens/dna/). The GRCh38 gencode v22 CTAT transcriptome library is available through the Broad Institute (https://data.broadinstitute.org/Trinity/CTAT_RESOURCE_LIB/). Raw sequencing data is available through the European Genome-Phenome Archive (EGA) under accession ID EGAS50000000164.

Research involving human participants, their data, or biological material

Policy information about studies with human participants or human data. See also policy information about sex, gender (identity/presentation), and sexual orientation and race, ethnicity and racism.

Reporting on sex and gender

Biological sex was considered in the study design. Biological sex was determined based on self-reporting. The disaggregated biological sex data is presented in the manuscript, and we obtained consent for sharing the individual-level biological sex data. Out of the 25 patients enrolled, 40% were female (10 patients) and 60% were male (15 patients).

Reporting on race, ethnicity, or other socially relevant groupings

Ethnicity and race were considered in the study design and were provided by the participants and was based on self-reporting. Participants were asked if they identified as one of the following: American Indian or Alaska Native, Asian, Native Hawaiian or Pacific Islander, Black or African American, White or Caucasian or Other. Participants were able to choose more than one race/ethnicity and were also given the option not to answer. Further, participants were asked if they identified as Hispanic or Latino. Out of the 25 patients enrolled, 3 patients (12%) were Black or African American, 17 patients (68%) were Hispanic (16 White or Caucasian Hispanic [64%], 1 Mestizo [4%]), and 5 patients (20%) were Non-Hispanic White.
However, because race/ethnic demographic data was not used as inclusion criteria and did not serve as a factor in clinical intervention, they are not confounding variables in our analysis. Ultimately our clinical trial results are interpreted as a series of n = 1 studies.

Population characteristics

Patients with solid malignancies (19 of 25 enrolled, 76%) and hematological malignancies (6 of 25 enrolled, 24%) were enrolled. A total of 12 different pediatric malignant diseases were included: acute lymphoblastic leukemia (3 patients), acute myeloid leukemia (3 patients), astrocytoma (1 patient), ependymoma (1 patient), Ewing's sarcoma (4 patients), glioblastoma (1 patient), malignant rhabdoid tumor (1 patient), medulloblastoma (1 patient), neuroblastoma (1 patient), osteosarcoma (4 patients), rhabdomyosarcoma (4 patients) and Wilms' tumor (1 patient) (Figure 2d). Overall, all hematologic malignancies were leukemias (three acute lymphoblastic leukemia and three acute myeloid leukemia, 12% each), while solid malignancies consisted of sarcoma (48%), central nervous system (CNS) tumors (20%), and kidney cancers (8%) (Figure 2). The median age of the patient cohort was 10 years of age (minimum age 0.81 years, maximum age 19 years).

Recruitment

Patients were recruited from the patient population at Nicklaus Childrens Hospital with whom the study investigators already had an existing relationship with, or from referrals from other physicians. Investigators recruited patients who fit the inclusion criteria, particularly that of having suspected or confirmed diagnosis of recurrent or refractory cancer, and who could not be excluded due to having malignant tissue unavailable or insufficient for testing or whose cancers had a >90% cure rate with safe standard therapy. Due to this exclusion criteria, investigators may have had a potential bias against patients who might be reasonably treated with standard therapies.

Ethics oversight

The study IRB protocol was submitted by Nicklaus Children's Hospital and received approval by WCG IRB (WIRB and Copernicus Group IRB), IRB number 20181421. IRB-reliance was approved by Florida International University's IRB.

Note that full information on the approval of the study protocol must also be provided in the manuscript.

Field-specific reporting

Please select the one below that is the best fit for your research. If you are not sure, read the appropriate sections before making your selection.

☒ Life sciences ☐ Behavioural & social sciences ☐ Ecological, evolutionary & environmental sciences

For a reference copy of the document with all sections, see nature.com/documents/nr-reporting-summary-flat.pdf

Life sciences study design

All studies must disclose on these points even when the disclosure is negative.

Sample size

The primary endpoint of this study is providing pediatric cancer patients with access to personalized treatment options through Functional Precision Medicine, defined as drug sensitivity testing (DST) data and/or genomics data in a clinically-actionable time frame (within 4 weeks), with a null hypothesis of <30% of patients receiving FPM data and meeting the endpoint.
To test this hypothesis, a one-sided exact binomial test was applied with an alpha level of 0.025. To achieve at least 90% power, the null

	hypothesis will be rejected when at least 16 out of 25 patients receive FPM data within 4 weeks on the study. With that outcome, we would have 95% confidence that the true feasibility rate is at least 30% (95% CI: 0.425, 1).<br>Correspondingly, 25 patients were determined as sufficient to provide appropriate statistical power for the primary study endpoint.
Data exclusions	Data from patients were excluded if rapid disease progression resulted in the patient passing away prior to return for Molecular Tumor Board discussion of ex vivo drug sensitivity testing (DST) results.
Replication	Due to the limited sample available from each patient, and to return results in a clinically relevant timeframe to allow for treatment decisions to be made, ex vivo drug sensitivity testing (DST) was only performed once for each patient. Tissue quantity limitations are known technical challenges in personalized cancer medicine studies, which often limits experimental replication. Technical replicates and positive and negative controls for drug sensitivity testing were included on each plate. Additionally, when PDC material was available and combination testing was requested, we performed DST repeated testing. The tissue limitations limiting replicates also impacted the number of experiments that were performed for PDC validation studies, including genomic/transcriptomic analysis and immunofluorescence analysis. However, multiple validation approaches were used on the same sample affirming the biological relevance of our PDC models.
Randomization	This is a prospective observational cohort study and therefore, randomization is not applicable.
Blinding	Blinding was not relevant because this is a prospective observational cohort study

Behavioural & social sciences study design

All studies must disclose on these points even when the disclosure is negative.

Study description	*Briefly describe the study type including whether data are quantitative, qualitative, or mixed-methods (e.g. qualitative cross-sectional, quantitative experimental, mixed-methods case study).*
Research sample	*State the research sample (e.g. Harvard university undergraduates, villagers in rural India) and provide relevant demographic information (e.g. age, sex) and indicate whether the sample is representative. Provide a rationale for the study sample chosen. For studies involving existing datasets, please describe the dataset and source.*
Sampling strategy	*Describe the sampling procedure (e.g. random, snowball, stratified, convenience). Describe the statistical methods that were used to predetermine sample size OR if no sample-size calculation was performed, describe how sample sizes were chosen and provide a rationale for why these sample sizes are sufficient. For qualitative data, please indicate whether data saturation was considered, and what criteria were used to decide that no further sampling was needed.*
Data collection	*Provide details about the data collection procedure, including the instruments or devices used to record the data (e.g. pen and paper, computer, eye tracker, video or audio equipment) whether anyone was present besides the participant(s) and the researcher, and whether the researcher was blind to experimental condition and/or the study hypothesis during data collection.*
Timing	*Indicate the start and stop dates of data collection. If there is a gap between collection periods, state the dates for each sample cohort.*
Data exclusions	*If no data were excluded from the analyses, state so OR if data were excluded, provide the exact number of exclusions and the rationale behind them, indicating whether exclusion criteria were pre-established.*
Non-participation	*State how many participants dropped out/declined participation and the reason(s) given OR provide response rate OR state that no participants dropped out/declined participation.*
Randomization	*If participants were not allocated into experimental groups, state so OR describe how participants were allocated to groups, and if allocation was not random, describe how covariates were controlled.*

Ecological, evolutionary & environmental sciences study design

All studies must disclose on these points even when the disclosure is negative.

Study description	*Briefly describe the study. For quantitative data include treatment factors and interactions, design structure (e.g. factorial, nested, hierarchical), nature and number of experimental units and replicates.*
Research sample	*Describe the research sample (e.g. a group of tagged Passer domesticus, all Stenocereus thurberi within Organ Pipe Cactus National Monument), and provide a rationale for the sample choice. When relevant, describe the organism taxa, source, sex, age range and any manipulations. State what population the sample is meant to represent when applicable. For studies involving existing datasets, describe the data and its source.*
Sampling strategy	*Note the sampling procedure. Describe the statistical methods that were used to predetermine sample size OR if no sample-size calculation was performed, describe how sample sizes were chosen and provide a rationale for why these sample sizes are sufficient.*
Data collection	*Describe the data collection procedure, including who recorded the data and how.*
Timing and spatial scale	*Indicate the start and stop dates of data collection, noting the frequency and periodicity of sampling and providing a rationale for*

Timing and spatial scale	*these choices. If there is a gap between collection periods, state the dates for each sample cohort. Specify the spatial scale from which the data are taken*
Data exclusions	*If no data were excluded from the analyses, state so OR if data were excluded, describe the exclusions and the rationale behind them, indicating whether exclusion criteria were pre-established.*
Reproducibility	*Describe the measures taken to verify the reproducibility of experimental findings. For each experiment, note whether any attempts to repeat the experiment failed OR state that all attempts to repeat the experiment were successful.*
Randomization	*Describe how samples/organisms/participants were allocated into groups. If allocation was not random, describe how covariates were controlled. If this is not relevant to your study, explain why.*
Blinding	*Describe the extent of blinding used during data acquisition and analysis. If blinding was not possible, describe why OR explain why blinding was not relevant to your study.*

Did the study involve field work? ☐ Yes ☐ No

Field work, collection and transport

Field conditions	*Describe the study conditions for field work, providing relevant parameters (e.g. temperature, rainfall).*
Location	*State the location of the sampling or experiment, providing relevant parameters (e.g. latitude and longitude, elevation, water depth).*
Access & import/export	*Describe the efforts you have made to access habitats and to collect and import/export your samples in a responsible manner and in compliance with local, national and international laws, noting any permits that were obtained (give the name of the issuing authority, the date of issue, and any identifying information).*
Disturbance	*Describe any disturbance caused by the study and how it was minimized.*

Reporting for specific materials, systems and methods

We require information from authors about some types of materials, experimental systems and methods used in many studies. Here, indicate whether each material, system or method listed is relevant to your study. If you are not sure if a list item applies to your research, read the appropriate section before selecting a response.

Materials & experimental systems

n/a	Involved in the study	
☐	☒	Antibodies
☒	☐	Eukaryotic cell lines
☒	☐	Palaeontology and archaeology
☒	☐	Animals and other organisms
☐	☒	Clinical data
☒	☐	Dual use research of concern
☒	☐	Plants

Methods

n/a	Involved in the study	
☒	☐	ChIP-seq
☒	☐	Flow cytometry
☒	☐	MRI-based neuroimaging

Antibodies

Antibodies used	For immunofluorescence, the antibodies used were the following:<br>Anti-Nkx2.2 antibody, dil 1:50, Abcam, Catalog # ab191077, Clone (EPR14638), Lot number 1000528-2.<br>anti-β-Catenin antibody, dil 1:100, Cell Signaling, Catalog# 8480, clone (D10A8) Lot number 9.<br>anti-Desmin antibody, dil 1:100, Cell Signaling, Catalog# 5332, clone (D93F5), Lot number 4.<br>anti-MyoD1 antibody, dil 1:400, Cell Signaling, Catalog# 13812, clone (D8G3), Lot number 1.
Validation	The specificity of the antibodies purchased from commercial sources (Abcam and Cell Signaling) was validated by the manufacturer in-house.<br>Abcam: https://www.abcam.com/primary-antibodies/how-we-validate-our-antibodies<br>Antibody specificity is confirmed by looking at cells that either do or do not express the target protein within the same tissue. Initially, our scientists will review the available literature to determine the best cell lines and tissues to use for validation. We then check the protein expression by IHC/ICC/IF to see if it has the expected cellular localization. If the localization of the signal is as expected, this antibody will pass and is considered suitable for use in IHC/ICC/IF. We use a variety of methods, including staining multi-normal human tissue microarrays (TMAs), multi-tumor human TMAs, and rat or mouse TMAs during antibody development. These high-throughput arrays allow us to check many tissues at the same time, providing uniformly as all tissues are exposed to the exact same conditions.<br>Cell Signaling Technology: https://www.cellsignal.com/about-us/our-approach-process/antibody-validation-immunofluorescence

- Cell lines or tissues with known target expression levels are used to verify specificity.
- Appropriate cell lines and tissues are used to verify subcellular localization.
- Antibody performance is assessed on appropriate tissues.
- Cells are subjected to phosphatase treatment to verify phospho-specificity. Target specificity is also verified with the use of known knockout or null cell lines.
- Cells are subjected to siRNA treatment or over-expression of the target protein to verify target specificity.
- Activation state specification, target expression, and translocation are examined using ligands or inhibitors to modulate pathway activity.
- Requirement of threshold signal-to-noise ratio in antibody:isotype comparison and minimum fold-induction for phospho-specific antibodies ensures the greatest possible sensitivity.
- Fixation and permeabilization conditions are optimized; alternative protocols are recommended if necessary.
- Stringent testing ensures lot-to-lot consistency.

Eukaryotic cell lines

Policy information about cell lines and Sex and Gender in Research

Cell line source(s)	*State the source of each cell line used and the sex of all primary cell lines and cells derived from human participants or vertebrate models.*
Authentication	*Describe the authentication procedures for each cell line used OR declare that none of the cell lines used were authenticated.*
Mycoplasma contamination	*Confirm that all cell lines tested negative for mycoplasma contamination OR describe the results of the testing for mycoplasma contamination OR declare that the cell lines were not tested for mycoplasma contamination.*
Commonly misidentified lines (See ICLAC register)	*Name any commonly misidentified cell lines used in the study and provide a rationale for their use.*

Palaeontology and Archaeology

Specimen provenance	*Provide provenance information for specimens and describe permits that were obtained for the work (including the name of the issuing authority, the date of issue, and any identifying information). Permits should encompass collection and, where applicable, export.*
Specimen deposition	*Indicate where the specimens have been deposited to permit free access by other researchers.*
Dating methods	*If new dates are provided, describe how they were obtained (e.g. collection, storage, sample pretreatment and measurement), where they were obtained (i.e. lab name), the calibration program and the protocol for quality assurance OR state that no new dates are provided.*

☐ Tick this box to confirm that the raw and calibrated dates are available in the paper or in Supplementary Information.

Ethics oversight	*Identify the organization(s) that approved or provided guidance on the study protocol, OR state that no ethical approval or guidance was required and explain why not.*

Note that full information on the approval of the study protocol must also be provided in the manuscript.

Animals and other research organisms

Policy information about studies involving animals; ARRIVE guidelines recommended for reporting animal research, and Sex and Gender in Research

Laboratory animals	*For laboratory animals, report species, strain and age OR state that the study did not involve laboratory animals.*
Wild animals	*Provide details on animals observed in or captured in the field; report species and age where possible. Describe how animals were caught and transported and what happened to captive animals after the study (if killed, explain why and describe method; if released, say where and when) OR state that the study did not involve wild animals.*
Reporting on sex	*Indicate if findings apply to only one sex; describe whether sex was considered in study design, methods used for assigning sex. Provide data disaggregated for sex where this information has been collected in the source data as appropriate; provide overall numbers in this Reporting Summary. Please state if this information has not been collected. Report sex-based analyses where performed, justify reasons for lack of sex-based analysis.*
Field-collected samples	*For laboratory work with field-collected samples, describe all relevant parameters such as housing, maintenance, temperature, photoperiod and end-of-experiment protocol OR state that the study did not involve samples collected from the field.*
Ethics oversight	*Identify the organization(s) that approved or provided guidance on the study protocol, OR state that no ethical approval or guidance was required and explain why not.*

Note that full information on the approval of the study protocol must also be provided in the manuscript.

Clinical data

Policy information about clinical studies

All manuscripts should comply with the ICMJE guidelines for publication of clinical research and a completed CONSORT checklist must be included with all submissions.

Clinical trial registration	The trial registration number on clinicaltrials.gov is NCT03860376 [Ex Vivo Drug Sensitivity Testing and Mutation Profiling].
Study protocol	Study protocol can be provided but exclusively during the peer review process
Data collection	Data collection was performed at Nicklaus Children's Hospital, 3100 SW 62nd Avenue, Miami, Florida 33155 and the Florida International University, 11200 SW 8th Street, Miami, Florida 33199.
Outcomes	The primary endpoint of this study is patients receiving clinically-actionable treatment recommendations through Functional Precision Medicine, defined as drug sensitivity testing (DST) data and/or genomics data in a clinically-actionable time frame (within 4 weeks), with a null hypothesis of <30% of patients receiving FPM data and meeting the endpoint.<br>• To test this hypothesis, a one-sided exact binomial test will be applied with an alpha level of 0.025. To achieve at least 90% power, the null hypothesis will be rejected when at least 16 out of 25 patients receive FPM data within 4 weeks on the study. With that outcome, we would have 95% confidence that the true feasibility rate is at least 30% (95% CI: 0.425, 1).<br>The secondary endpoints of the study compare clinical impact of therapy selection through the use of FPM or through non-FPM guided (physician's choice) therapy.<br>Data underlying secondary endpoints can be reviewed in the Supplemental Materials – Clinical Outcomes.<br>Objective Response Rate<br>• Objective Response Rate (the percentage of responders among total evaluable patients) in the FPM guided cohort vs the conventional protocol cohort will be calculated. An Objective Response to treatment is defined as any patient who achieves either "Partial Response" or "Complete Response" as best overall response during the study period, with these response types determined by the individual physicians per standard guidelines for both solid cancers and hematological cancers (RECIST 1.1).<br>• Comparisons of the Objective Response to previous treatment and trial treatments (FPM-guided prior vs FPM-guided trial and conventional prior vs conventional trial) will be calculated using a two-sided McNemar's test for paired binary data with continuity correction.<br>• Comparison of Objective Response Rate during the trial between FPM-guided and conventional cohorts will be performed using Barnard's test.<br>Progression-Free Survival<br>• Hypothesis testing for differences in Progression-Free Survival (PFS) between FPM-guided and conventional therapy will be performed using a two-sample log-rank (Mantel-Cox) test.<br>• Hypothesis testing for differences in PFS between previous and trial regimens in both FPM-guided and conventional cohorts will be performed using Cox regression with clustered computation, due to the intracohort analysis representing repeated measures.<br>• Hypotheses testing for differences in Progression-Free Survival ratio between previous regimen and trial regimen (in both FPM-guided and conventional cohorts) will be performed using Wilcoxon matched pairs test.<br>Previous vs. Trial Progression-Free Survival Ratio<br>• Hypotheses testing for differences in incidence of Progression-Free Survival ratio ≥ 1.3x between previous regimen and trial regimen (in both FPM-guided and conventional cohorts) will be performed using Barnard's test

Dual use research of concern

Policy information about dual use research of concern

Hazards

Could the accidental, deliberate or reckless misuse of agents or technologies generated in the work, or the application of information presented in the manuscript, pose a threat to:

No	Yes	
☒	☐	Public health
☒	☐	National security
☒	☐	Crops and/or livestock
☒	☐	Ecosystems
☒	☐	Any other significant area

Experiments of concern

Does the work involve any of these experiments of concern:

No	Yes	
☒	☐	Demonstrate how to render a vaccine ineffective
☒	☐	Confer resistance to therapeutically useful antibiotics or antiviral agents
☒	☐	Enhance the virulence of a pathogen or render a nonpathogen virulent
☒	☐	Increase transmissibility of a pathogen
☒	☐	Alter the host range of a pathogen
☒	☐	Enable evasion of diagnostic/detection modalities
☒	☐	Enable the weaponization of a biological agent or toxin
☒	☐	Any other potentially harmful combination of experiments and agents

Plants

Seed stocks — *Report on the source of all seed stocks or other plant material used. If applicable, state the seed stock centre and catalogue number. If plant specimens were collected from the field, describe the collection location, date and sampling procedures.*

Novel plant genotypes — *Describe the methods by which all novel plant genotypes were produced. This includes those generated by transgenic approaches, gene editing, chemical/radiation-based mutagenesis and hybridization. For transgenic lines, describe the transformation method, the number of independent lines analyzed and the generation upon which experiments were performed. For gene-edited lines, describe the editor used, the endogenous sequence targeted for editing, the targeting guide RNA sequence (if applicable) and how the editor was applied.*

Authentication — *Describe any authentication procedures for each seed stock used or novel genotype generated. Describe any experiments used to assess the effect of a mutation and, where applicable, how potential secondary effects (e.g. second site T-DNA insertions, mosiacism, off-target gene editing) were examined.*

ChIP-seq

Data deposition

☐ Confirm that both raw and final processed data have been deposited in a public database such as GEO.

☐ Confirm that you have deposited or provided access to graph files (e.g. BED files) for the called peaks.

Data access links
May remain private before publication. — *For "Initial submission" or "Revised version" documents, provide reviewer access links. For your "Final submission" document, provide a link to the deposited data.*

Files in database submission — *Provide a list of all files available in the database submission.*

Genome browser session
(e.g. UCSC) — *Provide a link to an anonymized genome browser session for "Initial submission" and "Revised version" documents only, to enable peer review. Write "no longer applicable" for "Final submission" documents.*

Methodology

Replicates — *Describe the experimental replicates, specifying number, type and replicate agreement.*

Sequencing depth — *Describe the sequencing depth for each experiment, providing the total number of reads, uniquely mapped reads, length of reads and whether they were paired- or single-end.*

Antibodies — *Describe the antibodies used for the ChIP-seq experiments; as applicable, provide supplier name, catalog number, clone name, and lot number.*

Peak calling parameters — *Specify the command line program and parameters used for read mapping and peak calling, including the ChIP, control and index files used.*

Data quality — *Describe the methods used to ensure data quality in full detail, including how many peaks are at FDR 5% and above 5-fold enrichment.*

Software — *Describe the software used to collect and analyze the ChIP-seq data. For custom code that has been deposited into a community repository, provide accession details.*

Flow Cytometry

Plots

Confirm that:

- [ ] The axis labels state the marker and fluorochrome used (e.g. CD4-FITC).
- [ ] The axis scales are clearly visible. Include numbers along axes only for bottom left plot of group (a 'group' is an analysis of identical markers).
- [ ] All plots are contour plots with outliers or pseudocolor plots.
- [ ] A numerical value for number of cells or percentage (with statistics) is provided.

Methodology

Sample preparation	*Describe the sample preparation, detailing the biological source of the cells and any tissue processing steps used.*
Instrument	*Identify the instrument used for data collection, specifying make and model number.*
Software	*Describe the software used to collect and analyze the flow cytometry data. For custom code that has been deposited into a community repository, provide accession details.*
Cell population abundance	*Describe the abundance of the relevant cell populations within post-sort fractions, providing details on the purity of the samples and how it was determined.*
Gating strategy	*Describe the gating strategy used for all relevant experiments, specifying the preliminary FSC/SSC gates of the starting cell population, indicating where boundaries between "positive" and "negative" staining cell populations are defined.*

- [ ] Tick this box to confirm that a figure exemplifying the gating strategy is provided in the Supplementary Information.

Magnetic resonance imaging

Experimental design

Design type	*Indicate task or resting state; event-related or block design.*
Design specifications	*Specify the number of blocks, trials or experimental units per session and/or subject, and specify the length of each trial or block (if trials are blocked) and interval between trials.*
Behavioral performance measures	*State number and/or type of variables recorded (e.g. correct button press, response time) and what statistics were used to establish that the subjects were performing the task as expected (e.g. mean, range, and/or standard deviation across subjects).*

Acquisition

Imaging type(s)	*Specify: functional, structural, diffusion, perfusion.*
Field strength	*Specify in Tesla*
Sequence & imaging parameters	*Specify the pulse sequence type (gradient echo, spin echo, etc.), imaging type (EPI, spiral, etc.), field of view, matrix size, slice thickness, orientation and TE/TR/flip angle.*
Area of acquisition	*State whether a whole brain scan was used OR define the area of acquisition, describing how the region was determined.*

Diffusion MRI ☐ Used ☐ Not used

Preprocessing

Preprocessing software	*Provide detail on software version and revision number and on specific parameters (model/functions, brain extraction, segmentation, smoothing kernel size, etc.).*
Normalization	*If data were normalized/standardized, describe the approach(es): specify linear or non-linear and define image types used for transformation OR indicate that data were not normalized and explain rationale for lack of normalization.*
Normalization template	*Describe the template used for normalization/transformation, specifying subject space or group standardized space (e.g. original Talairach, MNI305, ICBM152) OR indicate that the data were not normalized.*
Noise and artifact removal	*Describe your procedure(s) for artifact and structured noise removal, specifying motion parameters, tissue signals and physiological signals (heart rate, respiration).*

Volume censoring | *Define your software and/or method and criteria for volume censoring, and state the extent of such censoring.*

Statistical modeling & inference

Model type and settings | *Specify type (mass univariate, multivariate, RSA, predictive, etc.) and describe essential details of the model at the first and second levels (e.g. fixed, random or mixed effects; drift or auto-correlation).*

Effect(s) tested | *Define precise effect in terms of the task or stimulus conditions instead of psychological concepts and indicate whether ANOVA or factorial designs were used.*

Specify type of analysis: ☐ Whole brain ☐ ROI-based ☐ Both

Statistic type for inference | *Specify voxel-wise or cluster-wise and report all relevant parameters for cluster-wise methods.*

(See Eklund et al. 2016)

Correction | *Describe the type of correction and how it is obtained for multiple comparisons (e.g. FWE, FDR, permutation or Monte Carlo).*

Models & analysis

n/a	Involved in the study
☐	☐ Functional and/or effective connectivity
☐	☐ Graph analysis
☐	☐ Multivariate modeling or predictive analysis

Functional and/or effective connectivity | *Report the measures of dependence used and the model details (e.g. Pearson correlation, partial correlation, mutual information).*

Graph analysis | *Report the dependent variable and connectivity measure, specifying weighted graph or binarized graph, subject- or group-level, and the global and/or node summaries used (e.g. clustering coefficient, efficiency, etc.).*

Multivariate modeling and predictive analysis | *Specify independent variables, features extraction and dimension reduction, model, training and evaluation metrics.*
